# The Comprehensive Analysis Identified an Autophagy Signature for the Prognosis and the Immunotherapy Efficiency Prediction in Lung Adenocarcinoma

**DOI:** 10.3389/fimmu.2022.749241

**Published:** 2022-04-22

**Authors:** Xizhe Li, Ziyu Dai, Xianning Wu, Nan Zhang, Hao Zhang, Zeyu Wang, Xun Zhang, Xisong Liang, Peng Luo, Jian Zhang, Zaoqu Liu, Yanwu Zhou, Quan Cheng, Ruimin Chang

**Affiliations:** ^1^ Department of Thoracic Surgery, Xiangya Hospital, Central South University, Changsha, China; ^2^ Hunan Engineering Research Center for Pulmonary Nodules Precise Diagnosis & Treatment, Changsha, China; ^3^ Department of Neurosurgery, Xiangya Hospital, Central South University, Changsha, China; ^4^ National Clinical Research Center for Geriatric Disorders, Changsha, China; ^5^ Department of Thoracic Surgery, The First Affiliated Hospital of University of Science and Technology of China (USTC), Division of Life Sciences and Medicine, University of Science and Technology of China, Hefei, China; ^6^ One-third Lab, College of Bioinformatics Science and Technology, Harbin Medical University, Harbin, China; ^7^ Department of Oncology, Zhujiang Hospital, Southern Medical University, Guangzhou, China; ^8^ Department of Interventional Radiology, The First Affiliated Hospital of Zhengzhou University, Zhengzhou, China

**Keywords:** lung adenocarcinoma, autophagy, gene signature, immune checkpoint therapy, TCGA, DRAM1

## Abstract

**Background:**

Lung adenocarcinoma (LUAD) is a fatal malignancy in the world. Growing evidence demonstrated that autophagy-related genes regulated the immune cell infiltration and correlated with the prognosis of LUAD. However, the autophagy-based signature that can predict the prognosis and the efficiency of checkpoint immunotherapy in LUAD patients is yet to be discovered.

**Methods:**

We used conventional autophagy-related genes to screen candidates for signature construction in TCGA cohort and 9 GEO datasets (tumor samples, n=2181; normal samples, n=419). An autophagy-based signature was constructed, its correlation with the prognosis and the immune infiltration of LUAD patients was explored. The prognostic value of the autophagy-based signature was validated in an independent cohort with 70 LUAD patients. Single-cell sequencing data was used to further characterize the various immunological patterns in tumors with different signature levels. Moreover, the predictive value of autophagy-based signature in PD-1 immunotherapy was explored in the IMvigor210 dataset. At last, the protective role of DRAM1 in LUAD was validated by *in vitro* experiments.

**Results:**

After screening autophagy-related gene candidates, a signature composed by CCR2, ITGB1, and DRAM1 was established with the ATscore in each sample. Further analyses showed that the ATscore was significantly associated with immune cell infiltration and low ATscore indicated poor prognosis. Meanwhile, the prognostic value of ATscore was validated in our independent LUAD cohort. GSEA analyses and single-cell sequencing analyses revealed that ATscore was associated with the immunological status of LUAD tumors, and ATscore could predict the efficacy of PD-1 immunotherapy. Moreover, *in vitro* experiments demonstrated that the inhibition of DRAM1 suppressed the proliferation and migration capacity of LUAD cells.

**Conclusion:**

Our study identified a new autophagy-based signature that can predict the prognosis of LUAD patients, and this ATscore has potential applicative value in the checkpoint therapy efficiency prediction.

## Introduction

Recent epidemiology studies have shown that lung cancer is the deadliest malignancy in the United States and China (1, 2). Lung adenocarcinoma (LUAD) is now holding the predominant position among all the pathological types of lung cancer. Although therapies of LUAD have achieved dramatic progress due to the innovation of surgery, chemotherapy, and targeted therapies, the prognosis of LUAD patients remains unsatisfactory (3). In the past decade, immune checkpoint blockade immunotherapies targeting PD-1 or other immune regulators are emerging as a new hope for LUAD patients (4, 5). However, few biomarkers can predict the efficacy of anti-PD-1 immunotherapy and stratify benefit population. Therefore, it is urgent to excavate more effective biomarkers to find appropriate patients that will benefit from anti-PD-1 immunotherapy.

Autophagy is a cellular process to degrade organelles and proteins by transporting them to the lysosomes, and its vital regulatory role in carcinogenesis is well known (6). During cancer development, autophagy may play tumor-promotor and tumor-suppressor roles in different cancers, and the specific function depends on the cancer type and development stage (7). Recent studies have shown that autophagy also has critical functions in tumor immunology (8). Like its double-edged sword functions in carcinogenesis, autophagy also plays a dual role in the anti-tumor immune response. On the one hand, autophagy activation may improve antigen presentation and immune recognition in dendritic cells (9, 10). On the other hand, autophagy can repress tumor-related antigen presentation by downregulation of MHC-I surface molecules (11). Meanwhile, pieces of evidence have revealed that autophagy in myeloid-derived suppressor cells (MDSCs) is associated with the M2 macrophage polarization and induces the immunosuppressive tumor microenvironment (12).

Since autophagy has a tight and elusive correlation with tumor immunology, researchers also pay attention to the potential association between autophagy and PD-1 blockade therapy. However, the effects of autophagy targeting drugs on PD-1 overexpressed cancer cells are controversial, and the interaction of autophagy regulators and PD-L1/PD-1 in LUAD is still to be revealed (13). Therefore, the investigation of a single regulator may not be enough to evaluate the full view of autophagy functions in LUAD. In comparison, the expression signature of autophagy-related genes may have the potential to predict the efficacy of anti-PD-1 immunotherapy in LUAD.

In this study, we selected 232 autophagy regulators and explored their expression in multiple datasets. Furthermore, we identified a signature (ATscore) composed of three autophagy-related genes, and this signature was correlated with the survival and tumor immunology factors of LUAD. Moreover, the prognostic predictive value of ATscores was validated in our independent LUAD cohort. The single-cell sequencing data analysis identified the potential relationship between this signature and the immune cell infiltration. And the ATscore could predict the therapeutic effects of the PD-1 blockade therapy. Therefore, our study provided a new predictive signature to evaluate the prognosis and possible effects of the PD-1 blockade therapy in LUAD.

## Materials and Methods

### Dataset Source and Preprocessing

**Figure 1:**
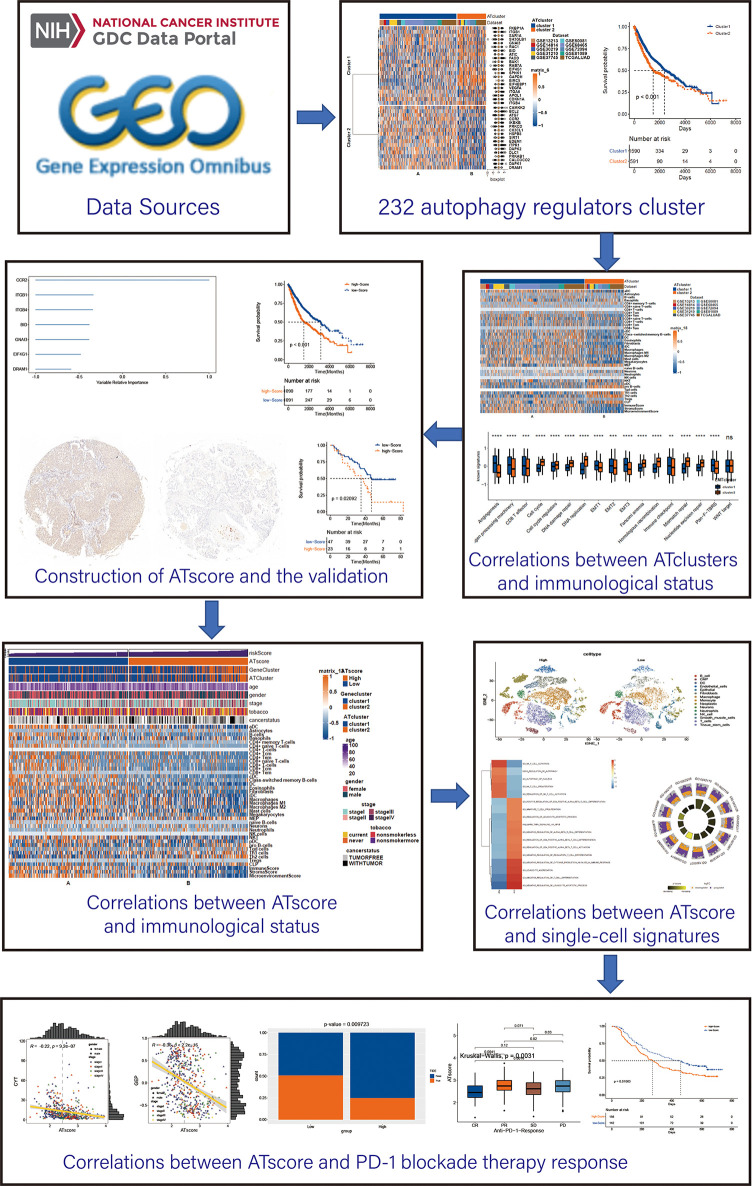
The workflow of the bioinforamtic analysis. Datasets were obtained from TCGA and GEO databases, and Consensus clustering were performed using autophagy regulators. After identifying the correlation between the autophagy clustering and immunological status, ATscore was constructed and proven to be a prognostic predictor of LUAD patients. The correlation between ATscore and immunological status of tumor was confirmed in datasets and single-cell sequencing data. Moreover, the predictor value of ATscore in PD-1blockade therapy response was confirmed.

The workflow of our study was summarized in **Figure 1**. Open LUAD gene expression datasets and corresponding clinical information were obtained from The Cancer Genome Atlas (TCGA) and Gene-Expression Omnibus (GEO) databases. Patients with incomplete clinicopathological information were removed in further analyses. In total, TCGA-LUAD dataset and 9 eligible GEO datasets (GSE13213, GSE14814, GSE30219, GSE31210, GSE37745, GSE50081, GSE68465, GSE72094, and GSE81089) were enrolled for further evaluation. The RNA-sequencing data were downloaded from GDC portal, and the fragments per kilobase million (FPKM) values were transformed into transcripts per kilobase million (TPM), so the data can be comparable between samples (14). The raw data of microarray datasets were gererated by Affymetrix or Agilent platforms. We used the RMA algorithm of the Affy software to perform the quaintly normalization and background correction for Affymetrix raw data. The consensus median polish algorithm in the Affymetrix software was used for summarizing of oligonucleotides for each transcript. Meanwhile, the R package “limma” was used to process the Agilent raw data. The signal intensity of TPM values from TCGA was similar with RMA-standardized values from microarray datasets.

### Consensus Clustering Analysis

We searched the canonical autophagy database HADb (Human Autophagy Database), and 232 autophagy regulators were identified to be autophagy signature clustering candidates (**Table S1**). Univariate COX analyses were performed to identify autophagy regulators for consensus clustering in the meta-cohort (P<0.001). Afterward, LUAD samples were grouped into clusters by consensus clustering analysis with the R package “ConsensusClusterPlus”. Kaplan‐Meier survival curves were created in each cluster, and log-rank tests were performed to compare the overall survival (OS) between subgroups. The “limma” package in R was used to investigate the differentially expressed genes (DEGs) between distinct autophagy clusters. The t-distributed stochastic neighbor embedding (t-SNE) analysis and the correlation analysis between autophagy regulators and DEGs were performed to validate the stability of the clustering.

### Functional Annotation Enrichment and Immune Cells Infiltration Analyses

To determine the differences of biological functions between sample subgroups, Gene Set Variation Analysis (GSVA), Gene Set Enrichment Analysis (GSEA), and Gene Ontology (GO) enrichment analysis were performed. The functions and pathways with a strict cut of the value of P < 0.05 were selected. For immune cell infiltrating estimation, we performed an xCell algorithm to quantify the proportion of the infiltrating immune cells in LUAD samples (15). Total 64 cell signatures were calculated, and 40 immune cell-related signatures were selected for display (aDC, Astrocytes, B-cells, Basophils, CD4+ memory T-cells, CD4+ naive T-cells, CD4+ T-cells, CD4+ Tcm, CD4+ Tem, CD8+ naive T-cells, CD8+ T-cells, CD8+ Tcm, CD8+ Tem, cDC, Class-switched memory B-cells, DC, Eosinophils, Fibroblasts, iDC, Macrophages, Macrophages M1, Macrophages M2, Mast cells, Megakaryocytes, MEP, naive B-cells, Neurons, Neutrophils, NK cells, NKT, pDC, pro B-cells, Tgd cells, Th1 cells, Th2 cells, Tregs, CLP, ImmuneScore, StromaScore and).

### Somatic Mutation Analyses

The copy number alterations (CNAs) and somastic mutations of LUAD patients was downloaded from the TCGA database. GSITIC analysis were applied to investigate the specific genomic event variations, and the threshold copy numbers at alteration peaks in different ATscore groups were illustrated. The somatic mutation analysis and mutation landscape delineation of TCGA was performed by the R package “maftool”.

### Generation of Autophagy Associated Signature

To construct the autophagy-based signature, the 38 autophagy regulators used for consensus clustering were subjected to univariate COX analysis in TCGA cohort., and those with P<0.05 were chosen as candidates for signature construction. To quantify the autophagy modification patterns of tumors, we use random survival forest analysis to perform dimension reduction to reduce noise or redundant genes among these prognostic autophagy regulators, and the relevant importance of each gene was calculated (14). Next, we conducted loop modeling to screen autophagy-based signatures based on the importance of each gene. The signature with the lowest P-value in Kaplan‐Meier analysis was identified as the autophagy-based signature, and the ATscore was calculated as follows:


ATscore = (−0.0923∗DRAM1 (geneexpressionlevel)) + 0.4621∗ITGB1 + (−0.2977∗CCR2)

The univariate and multivariate Cox regression analyses were used to investigate whether the ATscore can be an independent risk factor of OS for LUAD patients by R package “survival”.

### Single-Cell Characteristics Identification

For single-cell characteristics investigations, we obtained the single-cell RNA sequencing data of 49 clinical biopsies obtained from 30 patients (include 9 primary and 21 metastatic lung adenocarcinoma patients from an NCBI BioProject #PRJNA591860. The dataset was downloaded from ENA (European Nucleotide Archive (https://www.ebi.ac.uk/ena/browser/view/PRJNA591860). Follow genes were used as marker genes for the first clustering, immune (CD45+,*PTPRC*), epithelial/cancer (EpCAM+,*EPCAM*), stromal (CD10+,*MME*,fibo or CD31+,*PECAM1*,endo) (16). Cells previously annotated as epithelial cells were re-clustered using the following methods. We determined the single-cell copy number variation (CNV) through the “infercnv” package. We established an algorithm for the average expression of specific genes in each chromosome of all sample cells to distinguish between tumor and non-tumor epithelial cells (16). Infercnv searched cells with large copy number variations, sorted genes by chromosome position, and determined relative expression values by moving average method (17, 18). All epithelial cells as well as 300 fibroblasts and 300 endothelial cells were used as input. An additional 500 fibroblasts and 500 endothelial cells were used as reference controls. We scored the CNV degree for each cell and plotted the cells on a dendrogram, then cut at the highest point where all endothelial and fibroblasts belonged to a cluster (k = 6). All cells clustered with the spiked control were labelled as ‘non-tumor’, while the remaining two clusters were labelled as ‘tumor’. The non-tumor cells were annotated by “scCATCH” package. We further performed the cell clustering and dimension reduction by R package “Seurat”. Afterward, the principal component analysis (PCA), “FindNeighbors” package, and “FindClusters” package were used to construct the cell clustering. “UMAP” was used to visualize the expression profiling, and the “SingleR” package was used to cluster the non-malignant cells. The “FindCluster” function in “Seurat” package was used to identify genes that differentially expressed between two ATscore groups. GSVA and GSEA were performed to determine the functional annotation of ATscore. Statistical significance was set at |correlation coefficient| > 0.5 for GSVA, and FDR < 0.05 for GSEA (19). Furthermore, we performed the cell-cell interaction analysis by the “CellChat” package (20). The various receptor-ligand signaling expression modules between the high and low ATscore groups and the roles of different ATscore groups in specific molecular pathways were visualized.

### Immunotherapy and Molecular Therapy Response Prediction

To predict the potential immunotherapy response in patients with various levels of ATscores, T cell–inflamed gene expression profile (GEP), cytotoxic activity (CYT), and the Tumor Immune Dysfunction and Exclusion (TIDE) algorithm were performed in the TCGA cohort (21). To investigate the direct predictive value of ATscores on PD-1 therapy response, the IMvigor210 dataset was downloaded from http://research-pub.gene.com/IMvigor210CoreBiologies, and correlation between ATscore levels of patients and the anti-PD-1 therapy responses was calculated.

The chemotherapy response of LUAD patients from TCGA cohort was determined by the Genomics of Drug Sensitivity in Cancer database (GDSC, https://www. cancerrxgene.org). The IC50 values were calculated for the prediction of drug sensitivity by R package “pRRophetic”.

### Tissue Specimens and the Immunohistochemistry

The tissue sample collection in this study was approved by the ethics committee of Xiangya Hospital, Central South University (CSU; Changsha, China). Before the tumor sample collection, all patients were informed, and the written consent was obtained. From January 2011 to December 2012, 70 cases of LUAD tumor samples were collected from patients who underwent tumor resection at the Department of Thoracic Surgery, Xiangya Hospital, CSU. All patients did not receive radiotherapy or chemotherapy before the lung resection operation. The LUAD samples were collected from the edge of tumor lesions, and at least two experienced pathologists confirmed the pathological diagnosis. The collected samples were rapidly frozen in liquid nitrogen and transferred to a −80°C freezer for further assays. All patients were followed up every three months by telephone or a visit by our team for survival inquiry until death or the end of the follow-up.

IHC assays were performed for ATscore calculation in tissues. Paraffin-embedded tissues were cut into 4-μm sections. Sections were deparaffinized and boiled in 10 mM citrate buffer (pH 6.0) for antigen retrieval, and 3% H_2_O_2_ was used to block endogenous peroxidase activity. DRAM1 (sc-81713, Santacruz), CCR2 (ab203128, Abcam) and ITGB1 (ab134179, Abcam) antibodies were used as primary antibodies. The IHC staining scores were determined by combining staining intensity and the proportion of positively stained cells using histochemistry score (H-score). H-Score = (percentage of weak intensity cells ×1) + (percentage of moderate-intensity cells ×2) + (percentage of strong intensity cells ×3).

### Cell Culture and Transfection

A549 and H1299 cell lines were obtained from the Chinese Academy of Science Cell Bank (Shanghai, China), and cultured in RPMI‐1640 (Gibco) medium supplemented with 10% FBS (Gibco), 100 U/mL penicillin, and 100 μg/mL streptomycin (Gibco) in a humidified incubator with 5% CO2 at 37°C. The siRNA of DRAM1 (sense: CCUACAGUCCAUCAUCUCUUATT, antisense: UAAGAGAUGAUGGACUGUAGGTT) and the negative conthol (sense: UUCUCCGAACGUGUCACGUTT, antisense: ACGUGACACGUUCGGAGAATT) were obtained from Sangon Biotech (Shanghai, China), and transfected into cells by Lipofectamine 3000 (ThermoFisher) according to the manufacturer’s protocol.

### Western Blot Assays

The total protein of cell lines was extracted with RIPA lysis buffer (Beyotime) containing protease inhibitor for 20 min on ice. After the measurement of protein concentration by Bradford’s reagent (Beyotime), protein samples were separated by sodium dodecyl sulfate polyacrylamide gel electrophoresis (SDS-PAGE) and transferred onto polyvinylidene fluoride (PVDF) membranes. After blocking with 5% nonfat milk in TBST for 60 min, membranes were incubated with primary antibodies at 4°C overnight. DRAM1 (sc-81713, Santacruz) and GAPDH (GB11002, Servicebio) were used as primary antibodies. Anti-rabbit or anti-mouse IgG conjugated to horseradish peroxidase (HRP) (Proteintech) was used as the secondary antibody. Bands were visualized by Bio-Rad Image Lab Software.

### Cell Counting Kit-8 Assays

The proliferation ability of LUAD cells was monitored by cell counting kit-8 (Biosharp). A549 and H1299 cells (3*10^3^/well) were seeded in the 96-well plates. The Optical Density (OD_450_) were determined on 0, 24, 48, 72 and 96 h.

## 5-Ethylnyl-2′-Deoxyuridine Incorporation Assay

EdU assays were performed by kFluor488 Click-iT EdU Kit (KeyGEN biotech). A549 and H1299 cells (3000/well) were incubated in 95-well plates after the siRNA transfection. The EdU marking and the Hoechst 33342 identification were performed according to the manufacturer’s protocol.

### Colony Formation Assays

A549 and H1299 cells (1000/well) were seeded in the 6-well plates after the transfection. After 14 days of incubation, Paraformaldehyde fix solution (4% PFS) was used for the cells fixation, and 0.1% crystal violet (Beyotime Biotechnology) were used for identification of cell colonies.

### Transwell Assays

Transwell assays were performed to examine the migratory and invasive capacity of LUAD cells. For the transwell migration assay, 4*10^4^ cells in serum-free media were placed into the upper chamber of an insert (8 μm pore size, Corning). For the transwell invasion assay, 8*10^4^ cells in serum-free media were seeded into the upper chamber, which was precoated with Matrigel (Corning). For both transwell migration and invasion assays, medium with 20% FBS was added to the lower chamber. Cells were incubated for 24 h at 37°C, and nonmigrating cells were removed with cotton swabs. Migrated or invaded cells on the bottom of the membrane were fixed with 4% paraformaldehyde for 15 min and stained with crystal violet for 15 min. Then, stained cells were assessed by counting 5 random fields per chamber under a microscope.

### Statistical Analysis

R software (v3.6.3) was used for all statistical analyses. The normality of the variables was tested by the Shapiro-Wilk test. Differences between two normally distributed groups were determined by the Students’ t-test, and the Wilcoxon test measured differences between two non-normally distributed variables. One-way analysis of variance (ANOVA) tests was used as a parametric method for multiple groups comparison, while Kruskal–Wallis tests were used as a nonparametric method. For correlation coefficients calculation, Pearson correlation and distance correlation analyses were performed. Chi-square contingency tests were used for contingency tables analyses. After the ATscore calculation, R package sva was used for reducing the computational batch effect. Data visualization was performed using the R package ggplot2. Benjamini–Hochberg method was used for P values to FDRs conversion in the DEG analysis (22). ROC curves and the area under the curve (AUC) calculation were performed by R package timeROC. Kaplan‐Meier survival curves were created in each group, and log-rank tests were performed to compare the OS between subgroups. The univariate and multivariate Cox regression analyses were used to investigate the independent prognostic factor by R package survival. Survival curve visualization was performed by R package survminer. All heatmaps were generated by R package ComplexHeatmap. All the tests were two-sided, and P < 0.05 was considered statistically significant.

## Results

### The Consensus Clustering of Autophagy Regulators Predicted the Prognosis of LUAD Patients

**Figure 2:**
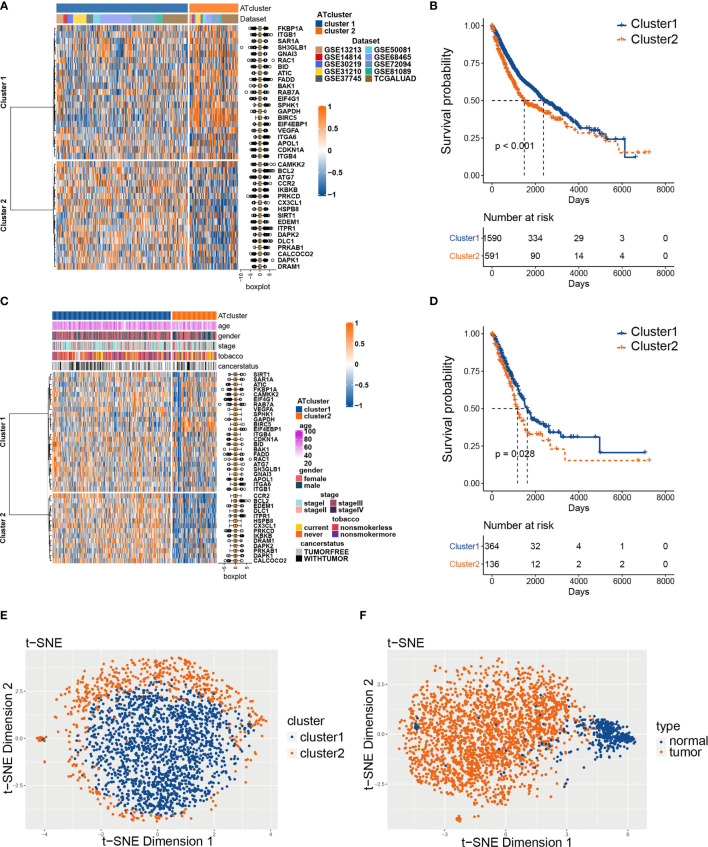
The consensus clustering of autophagy regulators predicted the prognosis of LUAD patients. The expression pattern of 38 autophagy regulators in the meta-cohort **(A)** and TCGA cohort **(C)**. The heatmaps showed upregulated genes (red) and downregulated genes (blue) of autophagy regulators in subgroups. The Kaplan‐Meier analysis showed that the clusters2 had worse prognosis than cluster1 in both meta-cohort **(B)** and TCGA cohort **(D)**. The t-SNE analyses showed that the two autophagy clusters were fine isolated **(E)**, and the autophagy-related regulators separated tumors from normal tissues noticeably **(F)**.

To explore the prognosis relevance of autophagy regulators, nine GEO datasets with available survival data (GSE13213, GSE14814, GSE30219, GSE31210, GSE37745, GSE50081, GSE68465, GSE72094, and GSE81089) and TCGA LUAD dataset were enrolled as the meta-cohort to construct the autophagy regulators consensus clustering. By the univariate COX analysis, 38 regulators with P<0.001 were selected for further analysis (**Table S2**). The R package ConsensusClusterPlus clusters the LUAD samples into subgroups based on the expression pattern of autophagy regulators (**Figure S1**). Our consensus matrix results showed that the LUAD meta-cohort could be distinctly divided into two subgroups, and these regulators exhibited different expression patterns in the meta-cohort and TCGA cohort (**Figures 2A, C**). Moreover, after we screening DEGs between clusters, the correlation analysis results showed that the clusters distinguished by autophagy regulators strongly correlated with identified DEGs (**Figure S2**).

Afterward, we examined whether the two subgroups of LUAD patients have different prognoses. The Kaplan‐Meier analysis results showed that cluster2 had a worse prognosis than cluster1 in the meta-cohort and TCGA cohorts (**Figures 2B, D**). Furthermore, we performed t-SNE to validate the subgroup assignment. Our results showed that the two clusters were fine isolated, and these 38 autophagy regulators separated tumors from normal tissues remarkably (**Figures 2E, F**).

### The Transcriptome Features of Autophagy Clusters

**Figure 3:**
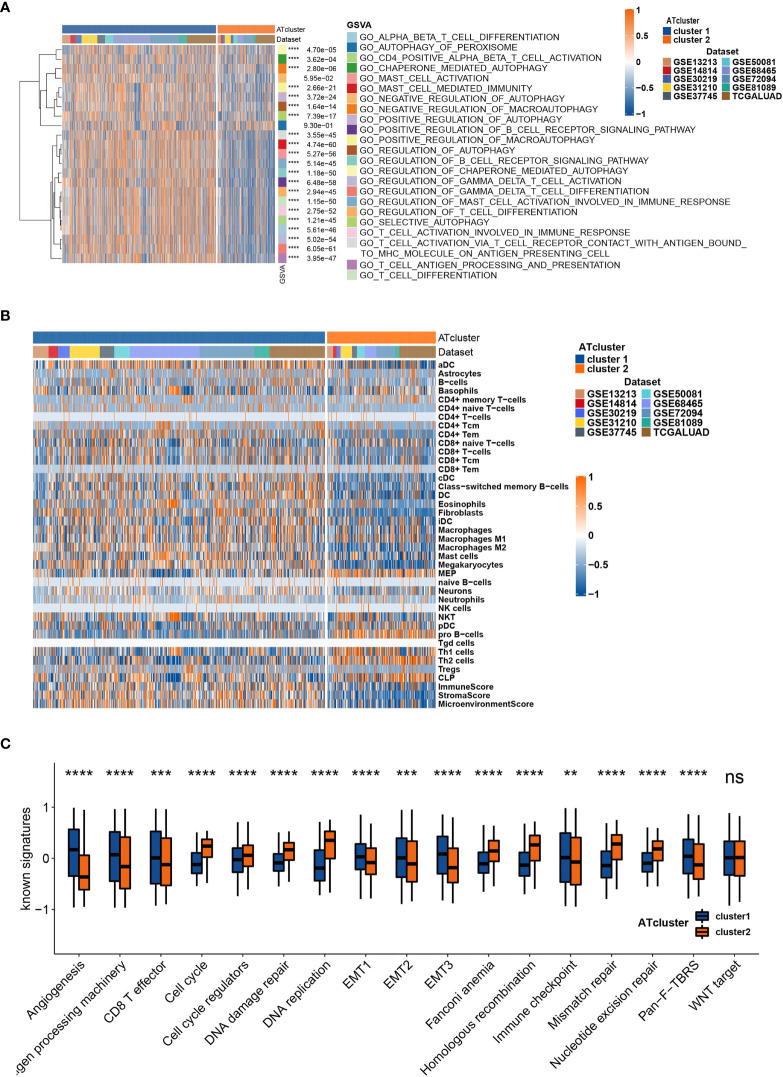
The transcriptome features of autophagy clusters in the meta-cohort. Unsupervised clustering assays showed that immune and autophagy-related biological functions were enriched in meta-cohort samples **(A)**, and the two autophagy clusters (ATcluster) have different immune infiltrating cells patterns **(B)**. The two autophagy clusters had different well-known signature patterns in the meta-cohort **(C)**. **P<0.01, ***P<0.001, ****P<0.0001. “ns” means no significance.

To determine the biological behavior differences between the two autophagy clusters (ATcluster), a GSVA enrichment analysis was performed to discover the potential function diversities. Interestingly, we found that plenty of immune-related and autophagy-associated pathways were enriched (**Figure 3A**). Therefore, we further investigated the unsupervised clustering of immune infiltrating cells in the meta-cohort. We found that the two ATclusters had different T cell-related infiltration clustering and significant discrepancy of tumor microenvironment scores (**Figure 3B**, **Figure S3A**), and these results were also confirmed in the TCGA cohort (**Figures S3B, C**). Moreover, the expression of immune-related genes in the two clusters had remarkable differences (**Figure S4**). Since T cells infiltration and the tumor microenvironment reshaping are the vital for PD-1 targeting therapies, these results suggested the potential association between our autophagy clustering and the response of PD-1 immune therapy. Moreover, the two autophagy clusters were associated with the well-known signatures in the meta-cohort and TCGA cohort (**Figure 3C**, **Figure S5A**). Apart from the immune infiltration, the autophagy clustering also correlates with proliferation-related genes and metabolic function of LUAD tumors (**Figures S5B, C** and **S6**).

### The Construction of Autophagy Modification Patterns and its Correlation With Prognosis of LUAD Patients and Functional Annotations

**Figure 4:**
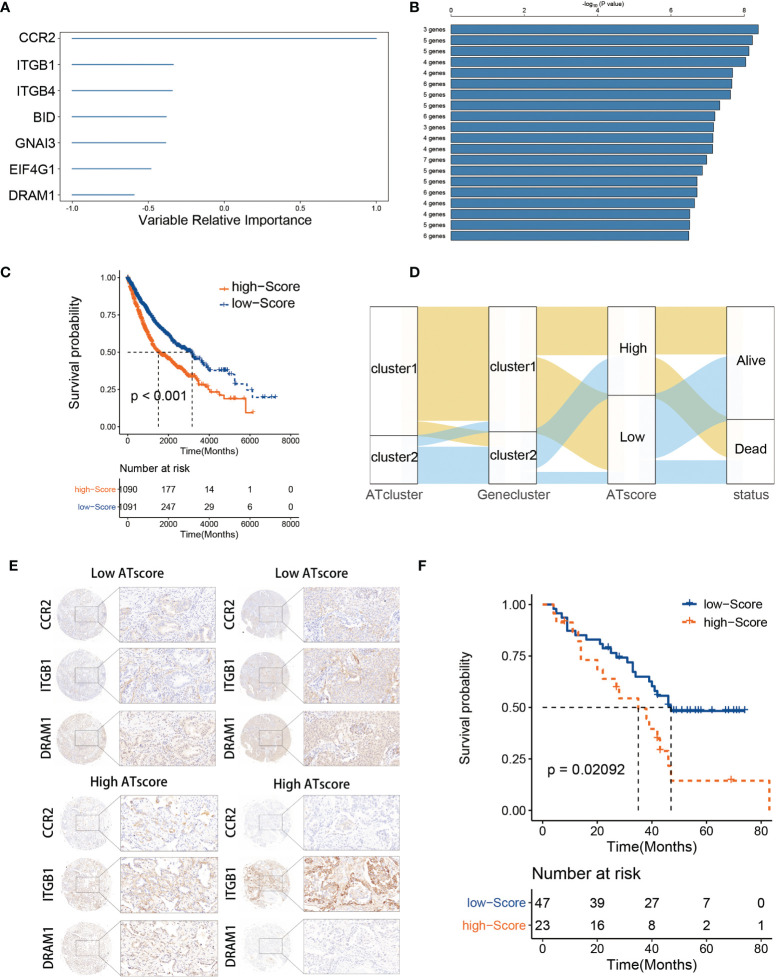
The construction of ATscore and the validation of its prognostic predictive value. The relative importance of the 20 autophagy regulators was calculated by random survival forest analysis, and the 7 most critical ones were showed **(A)**. The log-rank analyses showed that ATscore constructed by 3 genes predicted LUAD patients’ prognosis with a lowest P value **(B)**. LUAD patients with higher ATscore had noticeable worse overall survival in meta-cohort **(C)**. Sankey plot illustrating the interconnection among the ATclusters, Geneclusters, ATscores, and patient survival **(D)**. The representative IHC pictures of biopsies from independent cohort were showen **(E)**, and the prognostic predictive value of the ATscore was validated in independent cohort **(F)**.

The DEGs were acquired and adapted to investigate the comprehensive biological characteristics of two ATclusters (**Table S3**), and the consensus clustering by these DEGs was performed to construct the Geneclusters. Meanwhile, the 38 autophagy regulators used in the consensus clustering assay were selected for univariate COX analysis in TCGA cohort. The 20 regulators with P<0.05 were chosen as autophagy modification pattern candidates (**Table S4**). Furthermore, random survival forest analysis was used to explore the probability of autophagy modification pattern construction. After calculating the relative importance of 20 autophagy regulators, 7 genes were identified as the most critical factors (**Figure 4A**). Further log-rank analyses showed that the signature constructed by 3 genes (*CCR2*, *ITGB1*, and *DRAM1*) had the lowest P value and could predict LUAD patients’ prognosis with highest efficiency (**Figure 4B**). Therefore, we name this gene signature as the ATscore. Unsurprisingly, LUAD patients with higher ATscore had noticeable worse overall survival in meta-cohort (**Figure 4C**, **Figure S7A**), and the Sankey plot showed the interconnection among the ATclusters, Geneclusters, ATscores, and patient survival (**Figure 4D**). The ROC curve showed that ATscore can be a sensitive marker for 3-years overall survival of LUAD patients. Meanwhile, the AUC of ATscore was bigger than other reported models (**Figure S7B**) (23, 24). Moreover, both the univariate and multivariate COX regression analyses revealed that ATscore was an independent risk factor of LUAD patients (**Figure S7C**). Besides the LUAD cohorts, we also investigated the pan-cancer cohorts to explore whether the autophagy signature score can predict the survival of patients in other cancers. Our results showed that the predicted value of ATscore could be confirmed in multiple cancer cohorts (**Figure S8**). Taken together, our analyses constructed a new signature called ATscore, and this signature was correlated with the prognosis of LUAD patients and could act as an independent prognostic marker for LUAD patients.

To confirm the survival predictive value of ATscores in LUAD patients, we verified the prognostic value of ATscore in an independent validation cohort composed by 70 LUAD tumor samples from the Thoracic surgery department of Xiangya hospital. The IHC assays were performed to determine the expression levels of ATscore-related genes. Accordingly, the ATscores of these tumor samples were calculated. The samples with low expression levels of *CCR2* and *DRAM1* and high expression levels of *ITGB1* were classified into high ATscore group, vice versa (**Figure 4E**). The Kaplan‐Meier analysis results showed that patients with higher ATscores had significantly wrose prognosis than those with lower ATscores (**Figure 4F**). These results validated the prognostic predictive value of the ATscore in our LUAD cohort.

**Figure 5:**
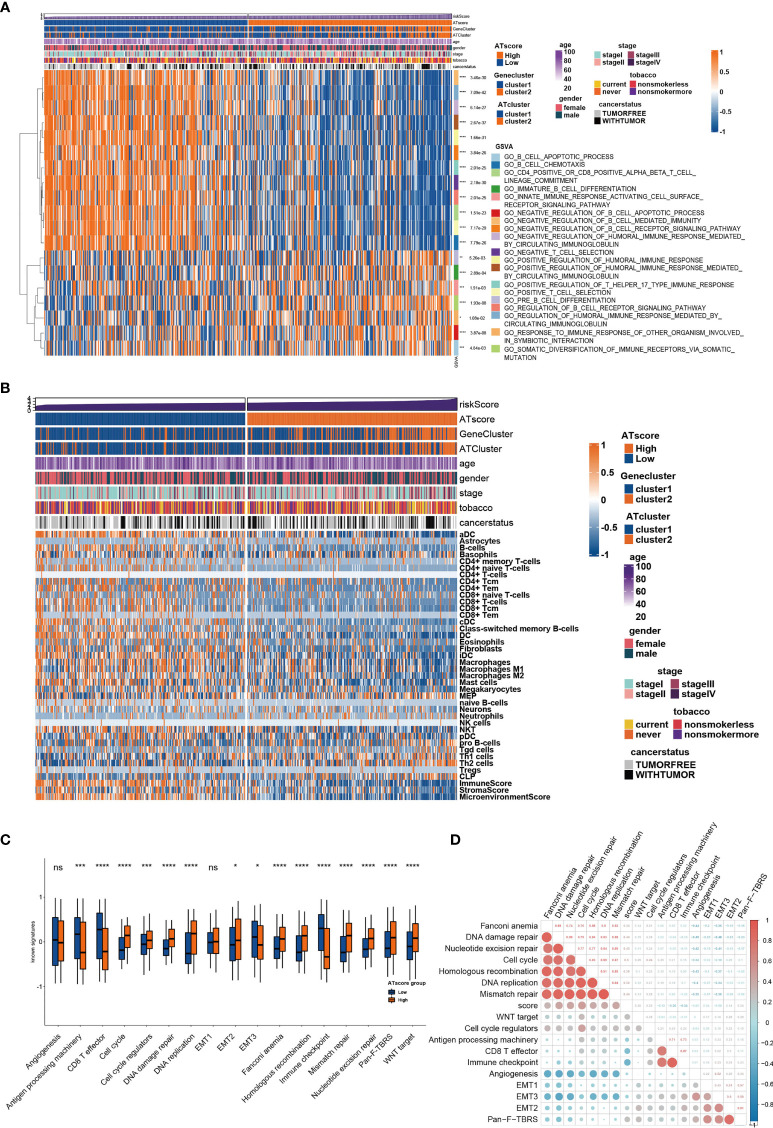
The transcriptome features of patients with various ATscores in TCGA cohort. The GSVA analysis showed the different immune-related patterns of patients with various ATscores in TCGA cohort **(A)**. The diversity of immune cells infiltrating patterns between patients with various ATscores was identified by xCell algorithm, and our results showed a more specific correlation of ATscores with checkpoints therapies-related immune cells **(B)**. Patients with various ATscores also presented different states of well-known signaturesand carcinogenesis pathways **(C, D)**. *P<0.05, **P<0.01, ***P<0.001, ****P<0.0001. “ns” means no significance.

To further investigate the functional traits of autophagy modification signature, transcriptome feature analyses were performed among patients with various ATscores. The GSVA analysis results showed that the ATscores were associated with T cell selection, T cell lineage commitment and other humoral immune-related pathways (**Figure 5A**). The further unsupervised clustering of immune infiltrating cells showed a more specific correlation of ATscores with checkpoints therapies-related immune cells (**Figure 5B**). Besides, we also found the relationship between the autophagy signature and the immune-related genes (**Figures S9**, **10**). Meanwhile, the results of CNA analysis revealed that patients with different ATscores had various copy number alterations (**Figure S11A**). Patients with high ATscore had higher levels of KRTAP9-9 (17q21.2) amplification and LCE3C (1q21.3) deletion, and those with low ATscore had higher levels of PLK2 (5q11.2) amplification. Somastic mutation analysis showed that patients with higher ATscore had higher mutation frequency of TP53 (55% vs. 41%), TTN (53% vs. 38%), MUC16 (45% vs. 34%), CSMD3 (44% vs. 30%) and RYR2 (43% vs. 29%) compared with those had lower ATscore (**Figure S11B**). Moreover, ATscores also presented a tight correlation with well-known signatures and carcinogenesis pathways (**Figures 5C, D**).

### The Correlation of ATScores With the Single-Cell Characteristics

**Figure 6:**
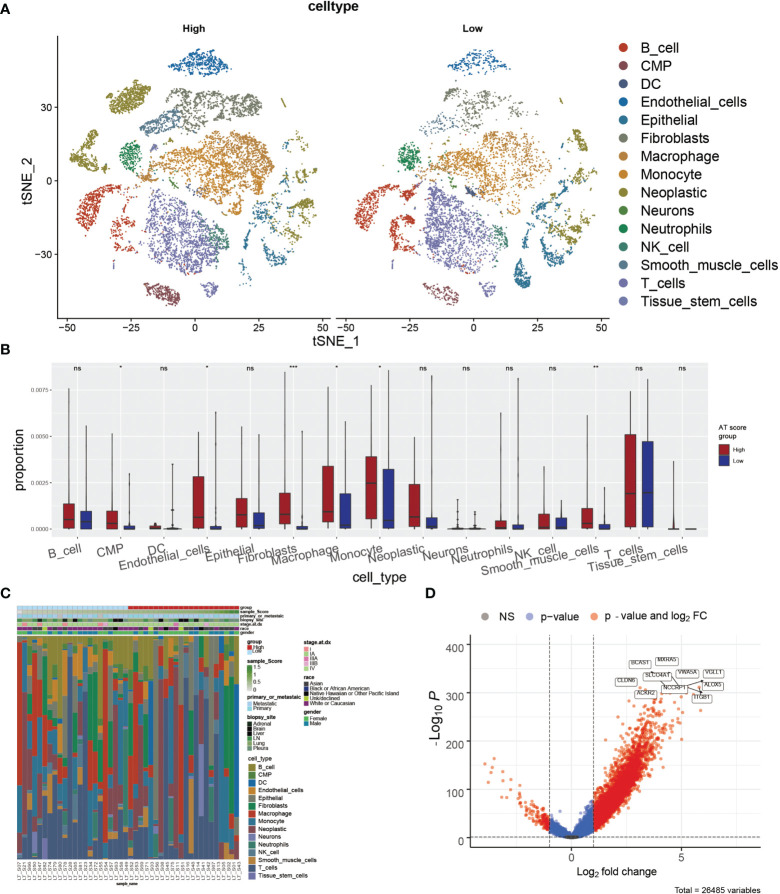
The correlation of ATscores with the single-cell characteristics. The single-cell patterns showed that tumor samples with higher ATscore had significantly more Neoplastic cells compared with low ATscore group **(A)**. The statistical analysis results of immune cells diversity assay showed that the tumor samples with lower ATscores had a lower proportion of common myeloid progenitor (CMP), macrophage, and monocyte than those with higher scores **(B)**, and the immune cells diversity between two ATscore groups was remarkable **(C)**. The DEGs between the single-cell samples with different levels of ATscores were visualized, and the most remarkable genes were identified **(D)**. *P<0.05, **P<0.01, ***P<0.001, ****P<0.0001. “ns” means no significance.

According to the potential correlation between T cell infiltrations of LUAD tumor and the autophagy signature, we further explored whether the ATscore could predict the single-cell characteristics by exploring the single-cell RNA sequencing data. We calculated the CNV of individual cells and the CNV score of each cell cluster by ‘infercnv’. The cells were classified into malignant and non-malignant cells (**Figures S12A, B**). Furthermore, the statistical analysis results showed that the tumor samples with lower ATscores had a lower proportion of common myeloid progenitor (CMP), macrophage, and monocyte than those with higher scores (**Figures 6A–C**). These results suggested the remarkable immune microenvironment diversity between the two ATscore groups.

**Figure 7:**
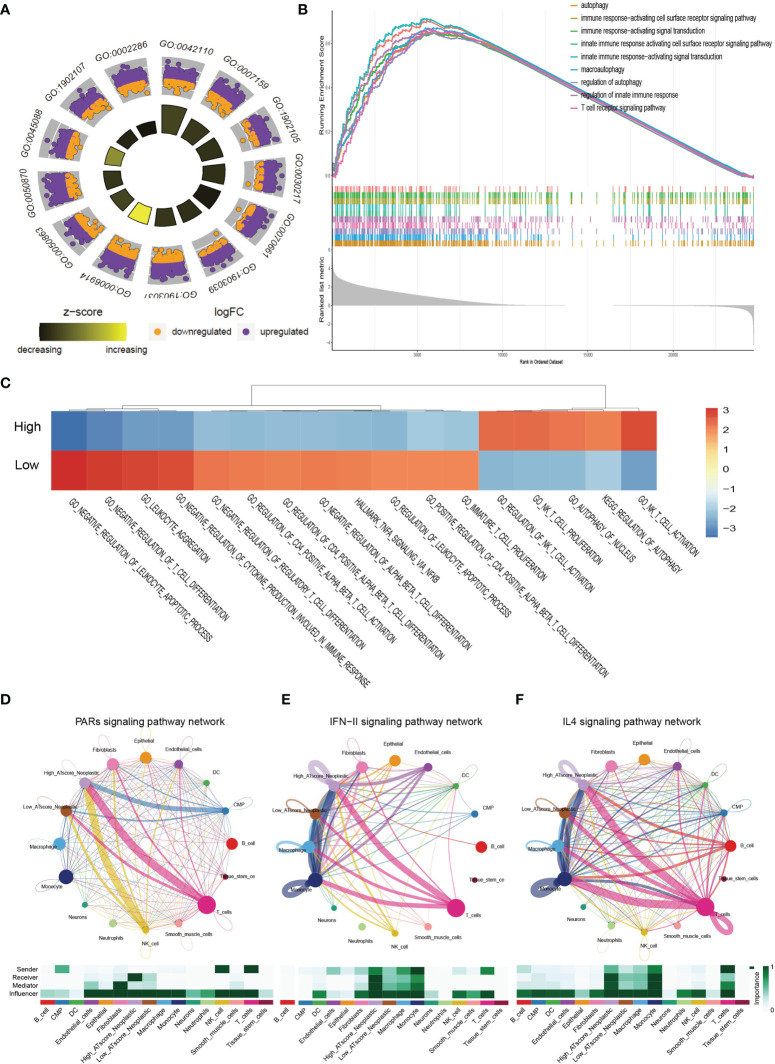
The correlation of ATscores with immune cells infiltrating and the cellular communication patterns. GO enrichment analysis **(A)**, GSEA **(B)** and GSVA **(C)** revealed that two ATscore groups had different autophagy and immune response-related cellular functions and signaling pathways. The cells with various ATscore levels have diverse tumor-related (PARs signaling) and immune response-related signaling pathway (IFN-II signaling and IL4 signaling) patterns **(D–F)**. Tumor cells with different ATscore levels play different roles in cellular interactions with tumor microenvironment cells.

We next focused on malignant cells with different ATscore. By the DEGs between the different levels of ATscores (**Figure 6D**), GO enrichment and GSEA assays were further performed. At the same time, we compared the different pathways between the two groups by GSVA analysis. Our results identified a remarkable difference between the two ATscore groups on the autophagy and immune response-related cellular functions and signaling pathways (**Figures 7A–C**).

Further, we explored the interaction between tumor cells with different ATscore levels and microenvironmental cells, and the results also showed a diversity of communication patterns between tumor cells with different ATscore levels (**Figures S12C, D**). It is well known that each cell has its unique communication pattern as receivers, senders, mediators, and influencers. Our results showed that T cells in the LUAD tumor microenvironment may communicate with malignant cells at high ATscore levels *via* PARs, IFN-II and IL-4 pathways, thereby regulating tumor cell proliferation and invasive capacity (**Figures 7D–F**).

### The Autophagy Modification Patterns Predicted Immunological Therapeutic Benefits

**Figure 8:**
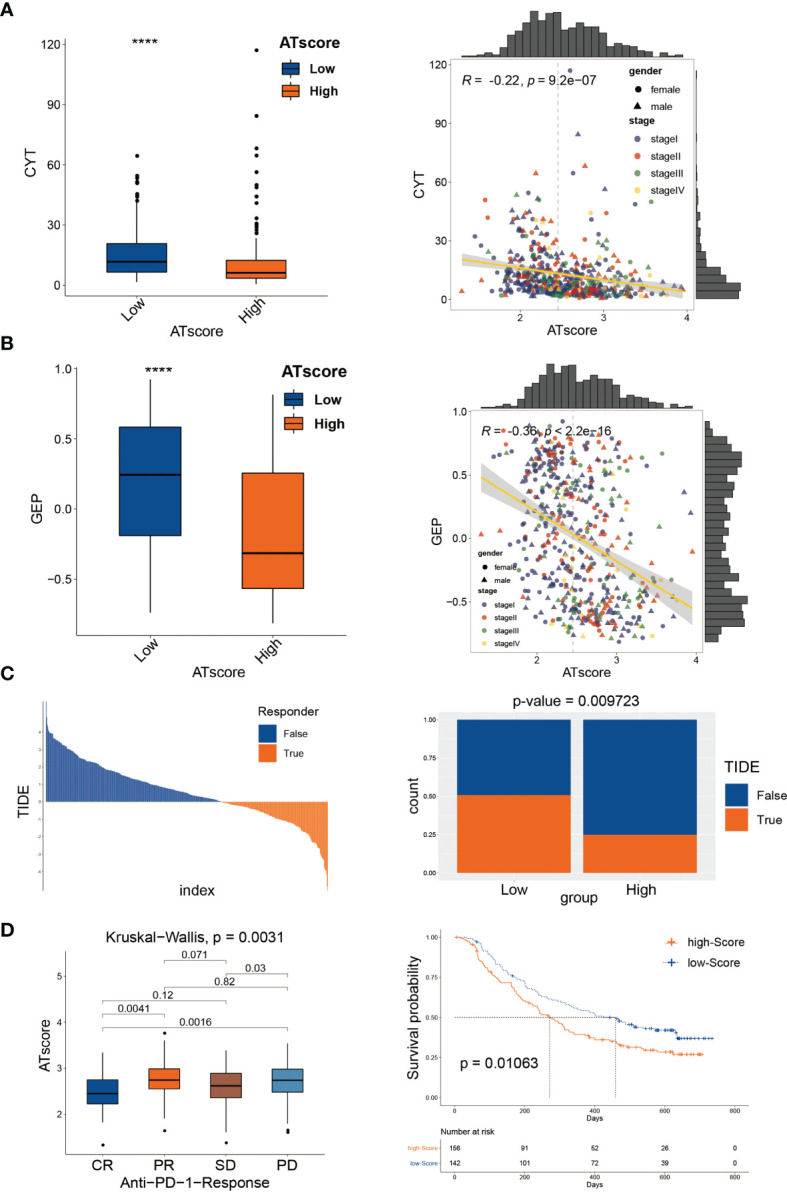
The autophagy signature predicted immunological therapeutic benefits. The patients with lower ATscore in the TCGA cohort had relatively higher CYT **(A)** and GEP **(B)** scores compared with those had higher ATscore. The results of TIDE algorithm showed that patients with higher ATscore in TCGA cohort might get poorer immunotherapy response than patients with lower ATscore **(C)**. In IMvigor210 cohort, patients with higher ATscores also had poorer anti-PD-1 response and prognosis than those with lower scores **(D)**. ****P<0.0001.

In the previous study, we have investigated the survival predictive role of ATscore in the LUAD cohort and explored the potential relationship between the ATscore and the immune escape of LUAD tumors. This section focused on the direct efficiency predictive value of ATscore in the PD-1 blockade therapy. Recently, T cell–inflamed GEP and CYT are emerging as predictive biomarkers for PD-1 blockade therapies (21). Therefore, we explored the correlation between ATscores and these well-known biomarkers. Our results showed that the patients with lower ATscore in the TCGA cohort had relatively higher GEP and CYT scores (**Figures 8A, B**). In addition, TIDE algorithm was also performed to evaluate the coherence of ATscores and the LUAD tumor immunotherapy response. Our results revealed that patients with higher ATscore in TCGA cohort might have poorer responses to immunotherapy (**Figure 8C**). These biomarker calculations suggested that the ATscores might have a negative correlation with the immunological therapy responses. Furthermore, we found that the levels of ATscores had a negative correlation with the PD-1 blockade therapy responses in the IMvigor210 cohort. In this cohort, patients with higher ATscores also had poorer anti-PD-1 response and prognosis than those with lower scores (**Figure 8D**). Moreover, we evaluated the chemotherapy response of LUAD patients with different ATscores. Our results showed that patients with higher ATscores had significantly higher IC50 value in many chemotherapy molecules compared with those with lower ATscore (**Figure S13**), especially in lung cancer sensitive AZD6244 and Gefitinib. These results indicated that patients with higher ATscores may also have poorer chemotherapy response than those with lower ATcores.

### DRAM1 Suppressed the Proliferation and Migration of LUAD Cells

**Figure 9:**
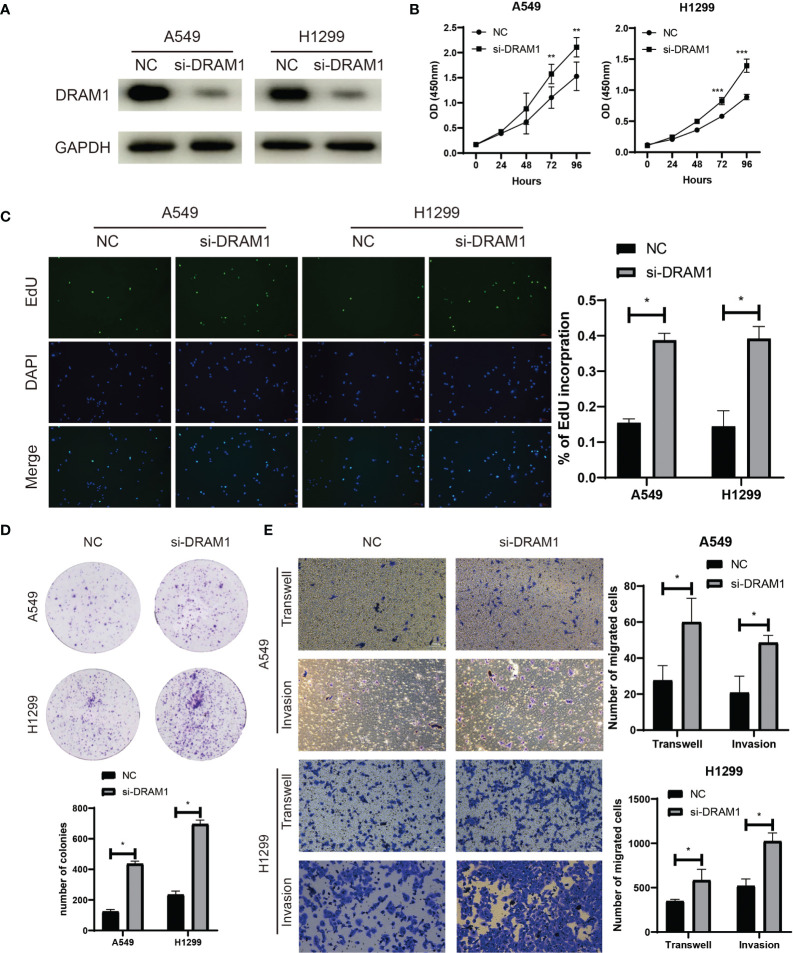
DRAM1 suppressed the proliferation and migration capacity of LUAD cells. The siRNA of DRAM1 significantly inhibited the expression of DRAM1 in LUAD cells **(A)**. The results of CCK-8 assays **(B)**, EdU assays **(C)**, and colony formation assays **(D)** revealed that the inhibiton of DRAM1 expression remarkablely promoted the proliferation and the DNA duplication of LUAD cells. The results of Transwell assays and Invasion assays showed that the impairment of DRAM1 expression enhanced the migration and invasion capacity of LUAD cells **(E)**. *P<0.05, **P<0.01, ***P<0.001.

Among the three autophagy regulators that make up the ATscore, *CCR2* and *ITGB1* were well-discussed and considered as vital regulators in the development of LUAD. However, the biological functions of *DRAM1* remained controversial. For validation of our predicted tumor suppressor role of *DRAM1* in the ATscore, we used siRNA to inhibited the expression of *DRAM1* in A549 and H1299 cells (**Figure 9A**). The CCK-8 assays, EdU assays and colony formation assays demonstrated that the inhibition of *DRAM1* significantly promoted the proliferation and DNA duplication ability of LUAD cells (**Figures 9B–D**). Meanwhile, the results of Transwell assays and invasion assays showed that the repression of *DRAM1* expression remarkably enhanced the migration and invasive capacity of LUAD cells (**Figure 9E**). These results demonstrated that *DRAM1* played as a tumor suppressor in LUAD cells, and its protective role in the ATscore was validated.

## Discussion

LUAD is one of the fatal malignancies globally, and anti-PD-1 immunotherapy is the frontier domain in the LUAD treatment. At present, how to identify patients who can get the best response to anti-PD-1 immunotherapy remains a challenge. Gene signatures are a kind of biological function pattern constructed by the expression data of multiple genes and can be used for prognostic and progression prediction in many types of malignancies (25, 26). Therefore, a signature which can both predict the prognosis and the response of PD-1 therapy in LUAD patient is urgent to be discovered. Recent studies have demonstrated that autophagy is involved in the innate immune response as a critical mechanism of immune receptor regulation. Consequently, PD-1 therapy biomarkers consisted of autophagy-related genes can be a new research direction. In this work, we constructed an autophagy-related signature with only three genes to predict the prognosis of LUAD patients and their response to PD-1 therapy, and the prognostic predictive value of this risk signature was validated in the independent LUAD cohort. A biomarker constructed with fewer genes in the clinical application means a lower cost and more accessible usage. Moreover, our results in pan-cancer cohorts inferred the potential application value of ATscore in other types of cancers. Therefore, the ATscore signature we constructed has good clinical application prospects in the future.

The tumor microenvironment (TME) is an emerging concept in cancer research. The conventional TME consists of extracellular matrix, cancer-associated fibroblasts, vascular epithelial cells, and infiltrating immune cells (27). In recent years, immune cell infiltration in solid tumors is a pivotal factor in the TME-related carcinogenesis mechanisms. Among the numerous infiltrated immune cells, polarized macrophages including M1 and M2 subtypeswere reported to play critical roles in TME (28). However, the immune infiltration pattern in most malignancies cannot be portrayed by a single type of cells. Other immune cells such as CD4+ T cells and Tregs should also be considered (29). Thus, building a gene signature that reflects the overall situation of immune cell infiltration is indispensable for evaluating immune-related therapies. Autophagy is proven to be a vital cellular process in immune cell regulation (30). Accordingly, the determination of autophagy pattern in primary tumors is promising for assessing immune cell infiltration in LUAD TME.

Previous studies constructed the autophagy-related signature to predict the prognosis of LUAD patients, but they only built the signature in TCGA dataset (31, 32) and verified in a few GEO datasets. A few of them focused on the autophagy-related long non-coding RNAs (lncRNAs) (33, 34). In Jie Zhu’s study, the authors used one GEO dataset to validate the prognosis predictive value of their signature (35). Therefore, the autophagy signatures constructed by these studies need more patient samples to verify their predictive value. In our study, we used TCGA dataset and 9 GEO datasets to construct the autophagy ATscore and validate its prognostic value. More than that, the predictive value of ATscore was validated in our independent LUAD cohort, and proven to be more efficient than other preported signatures (23, 24). Thus, the reliability and repeatability of our signature are more stable compared with other signatures constructed by previous studies. Moreover, the correlation between autophagy-based signatures and the immune checkpoint therapy efficacy was barely characterized. Herein, we used single-cell sequencing data analysis to explore the potential correlation between ATscores and the immune cell patterns, and immune-related pathway differences between various levels of ATscore were also identified. To further determine predictive value of ATscore in immune checkpoint immunotherapy, well-known algorithms such as TIDE, GEP, and CYT were used, and our results revealed remarkable correlations between ATscores and immunotherapy responses. Moreover, we used IMvigor210 dataset to directly confirm the PD-1 therapy response differences between patients with various ATscore levels, and the ATscores showed a noticeable negative correlation with the response of PD-1 therapy.

The biological role of three genes which were used in the ATscore calculation were well characterized in previous studies. CCR2 is a G protein-coupled receptor that binds the chemokine MCP-1 (CCL2) to recruit myeloid cells into the peripheral blood. Numerous studies have demonstrated that CCR2 participates in the development, especially the infiltration of the immune cells in lung cancer. It was demonstrated that CCR2 and CXCR1 signaling played a critical role in the cross-talk between tumor-associated macrophage and lung cancer cells (36). Recently, more studies revealed that CCR2 is involved in the ERα (Estrogen receptor α)-induced lung cancer cell invasion by macrophage infiltration mechanism and might be an essential component of cancer immunotherapy (37, 38). ITGB1 and DRAM1 were also abnormally expressed in many types of cancer including lung cancer (39–42). ITGB1 was a well-known oncogene and was found to have a critical role in the development of NSCLC. The expression of ITGB1 could be promoted by a long non-coding RNA ITGB1-DT, which was located in the opposite direction from the coding ITGB1 sequence (43). However, the biological functions of DRAM1 remained controversial. DRAM1 was found to be a p53 target gene and played a vital role in autophagy and apoptosis (44). Evidence revealed that DRAM1 could be a target of FTSJ1 and facilitate lung cancer progression (45). Interestingly, a few months later, another study showed that DRAM1 suppresses the development of lung cancer by promoting autophagy-related EGFR degradation (46). Herein, our *in vitro* experimental results showed that DRAM1 suppressed the proliferation and migration of LUAD cells. According to these data, the biological functions of component genes of ATscore were validated. Given the biological functions of these genes in lung cancer, they were also used to construct other tumor microenvironment-associated signatures (31, 32, 47, 48). These studies suggested the vital roles of these genes in developing cancers, and the specific biological functions of these genes should be identified in the future.

Taken together, we constructed a novel autophagy-based signature termed ATscore to predict the prognosis and anti-PD-1 immunotherapy efficacy in LUAD. Our study helps to elucidate the mechanism of LUAD progression and suggests a promising prognostic and therapeutic predictive model for LUAD patients.

## Data Availability Statement

The datasets presented in this study can be found in online repositories. The names of the repository/repositories and accession number(s) can be found in the article/**Supplementary Material**.

## Ethics Statement

The studies involving human participants were reviewed and approved by the ethics committee of Xiangya Hospital, Central South University. The patients/participants provided their written informed consent to participate in this study.

## Author contributions

Experimental performance and formal analysis, ZD, XW, NZ, HZ, ZW, XZ, XSL; funding acquisition, RC, YZ, XZL; project administration, QC; writing – original draft, XZL; writing – review & editing, QC, RC, PL, JZ, ZL. All authors contributed to the article and approved the submitted version.

## Funding

This work was supported by grants from Hunan Science and Technology Innovation Talent Program-Excellent Postdoctoral Innovation Talent Project (2020RC2010, 2021RC2029), China Postdoctoral Science Foundation Funded Project (2021M693558), Outstanding Youth Fund of Natural Science Foundation of Hunan Province (2021JJ20092), and Innovation-Driven Project of Central South University (2020CX043), and Hunan Provincial Natural Science Foundation Youth Project (2019JJ50941).

## Conflict of Interest

The authors declare that the research was conducted in the absence of any commercial or financial relationships that could be construed as a potential conflict of interest.

## Publisher’s Note

All claims expressed in this article are solely those of the authors and do not necessarily represent those of their affiliated organizations, or those of the publisher, the editors and the reviewers. Any product that may be evaluated in this article, or claim that may be made by its manufacturer, is not guaranteed or endorsed by the publisher.

## Abbreviations

ANOVA, one-way analysis of variance; ATcluster, autophagy cluster; CCL2, chemokine MCP-1; CMP, common myeloid progenitor; CNV, copy number variation; CYT, cytotoxic activity; DEGs, differentially expressed genes; ERα, Estrogen receptor α; FDRs, False discovery rates; GEO, gene-expression omnibus; GEP, gene expression profile; GO, gene ontology; GSEA, gene set enrichment analysis; GSVA, gene set variation analysis; HADb, human autophagy database; IHC, Immunohistochemistry; lncRNAs, long non-coding RNAs; LUAD, lung adenocarcinoma; MDSCs, myeloid-derived suppressor cells; MHC-I, major histocompatibility complex-I; OS, overall survival; PCA, the principal component analysis; PD-1, Programmed cell death protein 1; PD-L1, Programmed death-ligand 1; TCGA, The Cancer Genome Atlas; TIDE, Tumor Immune Dysfunction and Exclusion; TME, The tumor microenvironment; t-SNE, t-distributed stochastic neighbor embedding.
