# Supplementary material for: The Comprehensive Analysis Identified an Autophagy Signature for the Prognosis and the Immunotherapy Efficiency Prediction in Lung Adenocarcinoma
Source: Front Immunol. 2022 Apr 22;13:749241. doi: 10.3389/fimmu.2022.749241 (PMC9072793; doi:10.3389/fimmu.2022.749241)
Supplement: Supplementary file 1 [file DataSheet_1.pdf]

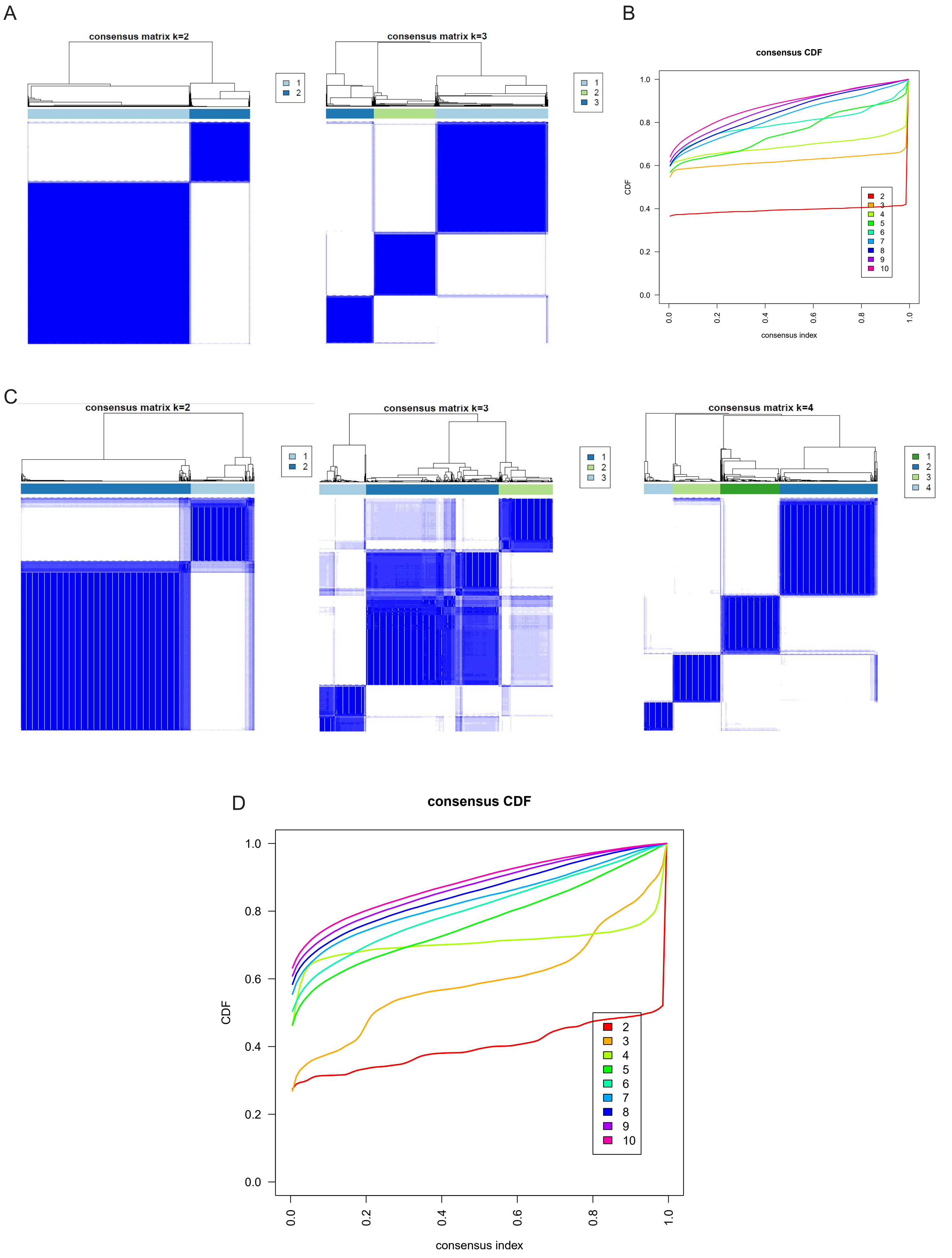

Figure S1: The consensus clustering based on the 38 autophagy regulators. The consensus clustering algorithm defining Lasso-gene clusters. Consensus matrixes for each k (k = 2–3 for meta-cohort (A) and k=2-4 for TCGA cohort (C)), displaying the clustering stability using 1000 iterations of hierarchical clustering. Consensus clustering cumulative distribution function (CDF) for k = 2–10 in meta-cohort (B) and TCGA cohort (D) were visualized.

A

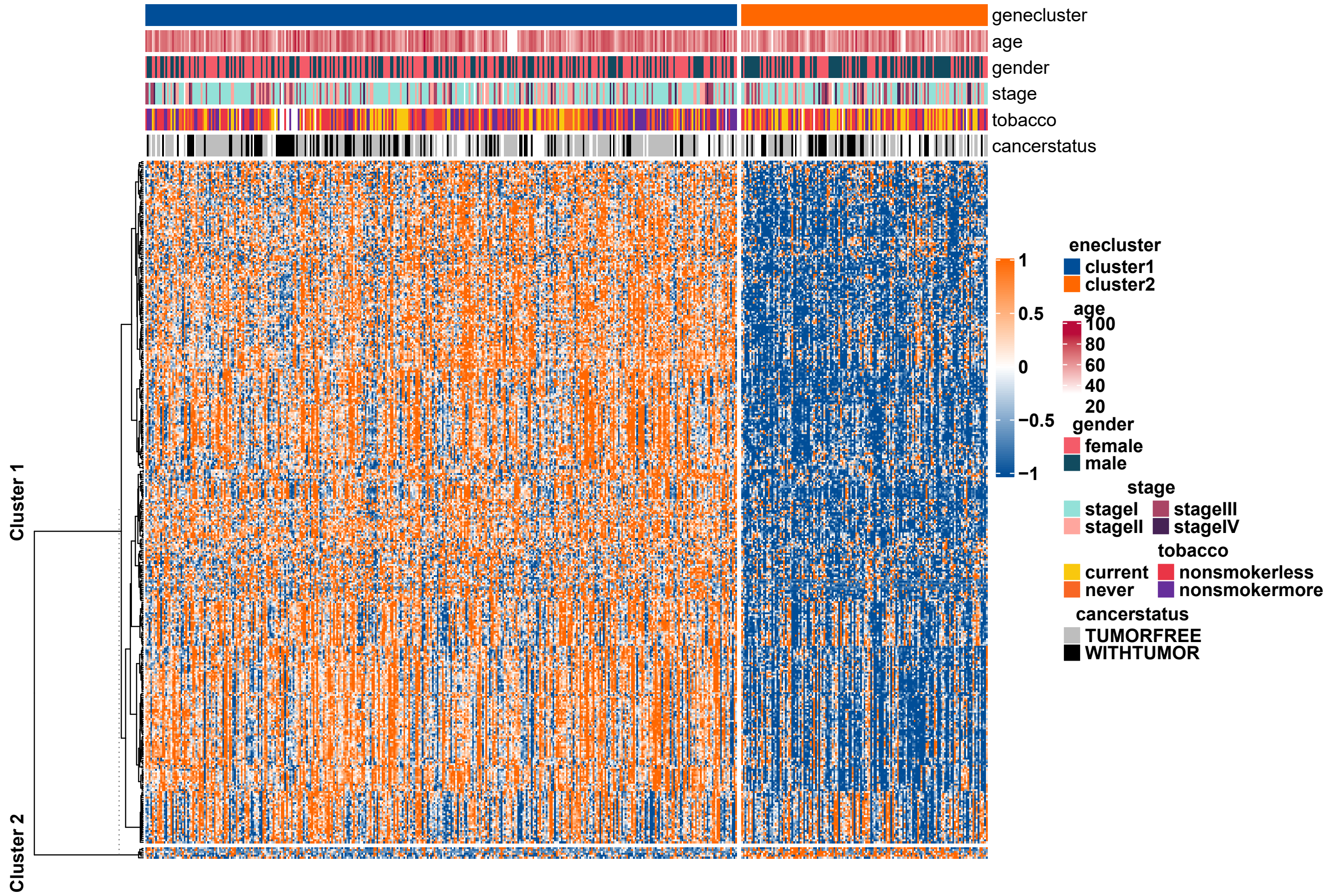

B

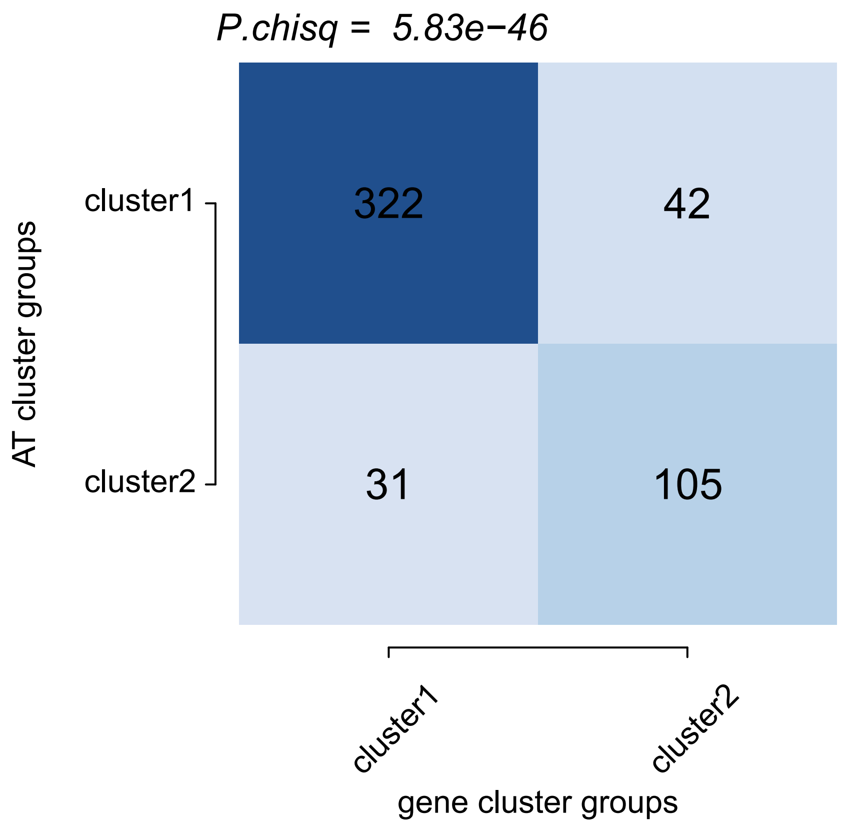

Figure S2: The expression pattern of genecluster was visualized (A). The heatmaps showed upregulated genes (red) and downregulated genes (blue) of DEGs in subgroups. The correlation analysis showed that the clusters distinguished by autophagy regulators strongly correlated with those by DEGs (B)

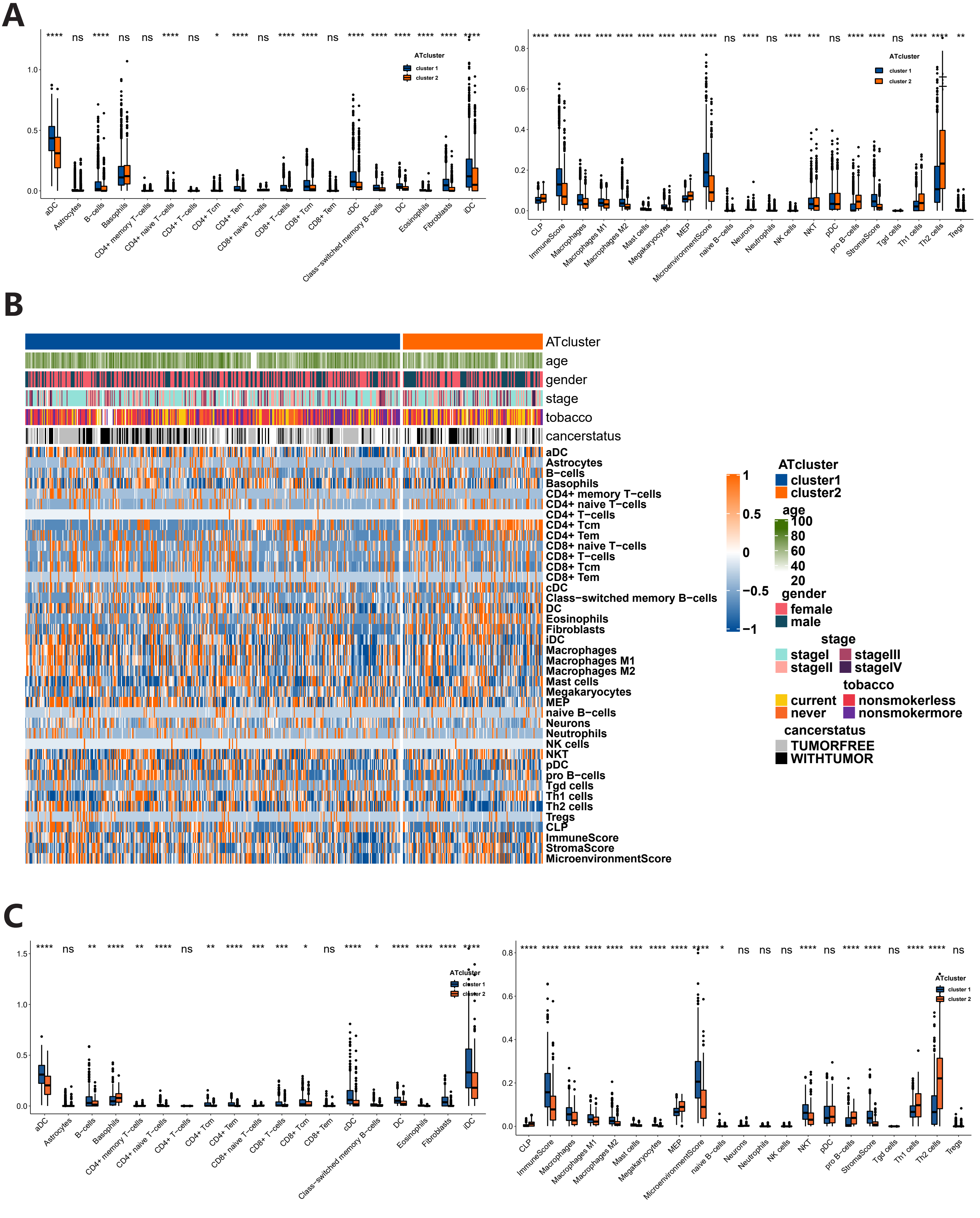

Figure S3: The correlation of ATclusters with immune cell infiltration in meta-cohort (A) and TCGA cohort was identified by xCell algorithm (B, C). The two subtypes had different immune infiltrating cells patterns. *P＜0.05, **P＜0.01, ***P＜0.001, ****P＜0.0001.

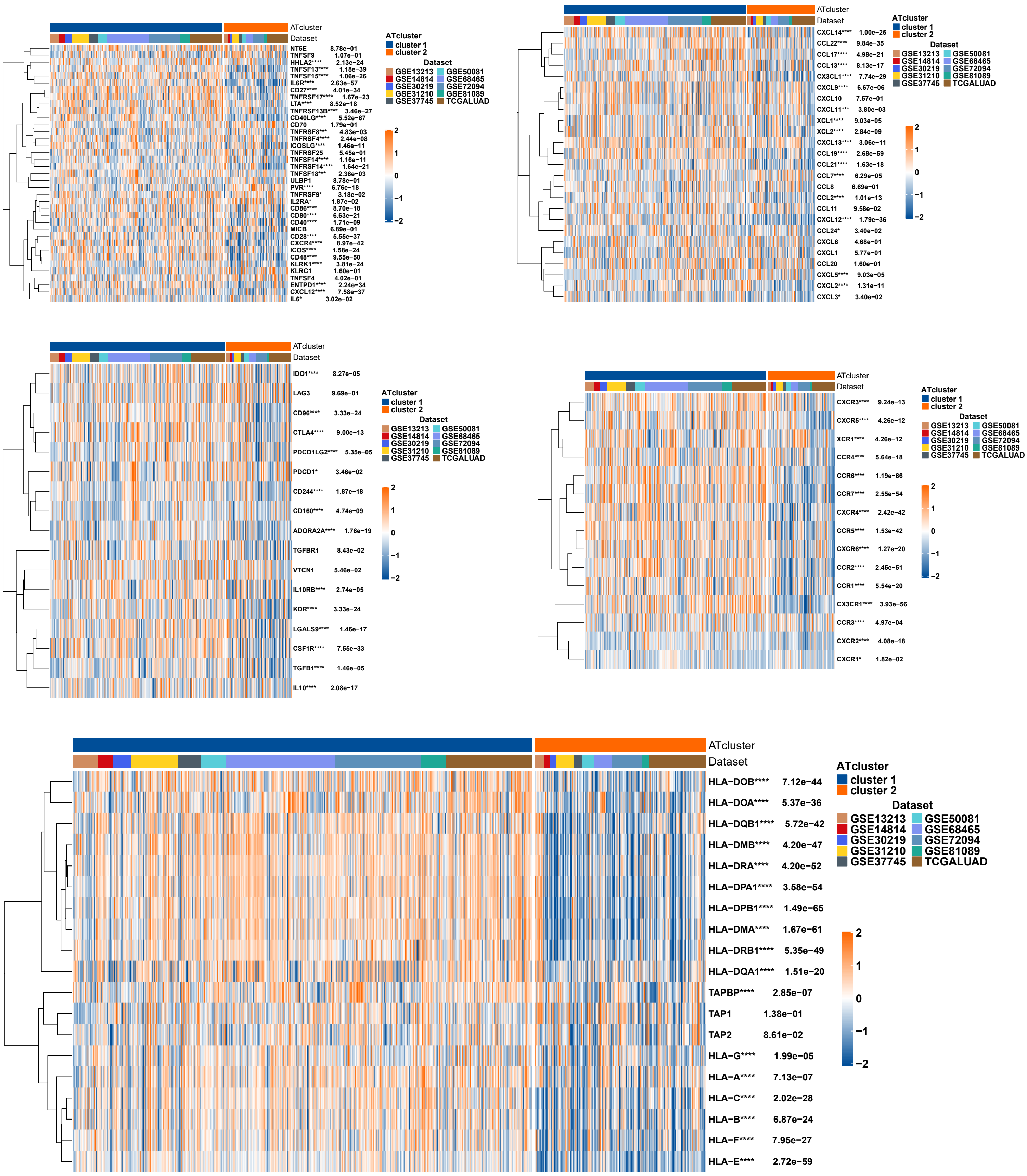

Figure S4: The Unsupervised clustering assays showed that different ATclusters had diverse expression levels of chemokines, receptors and HLA in meta-cohort.

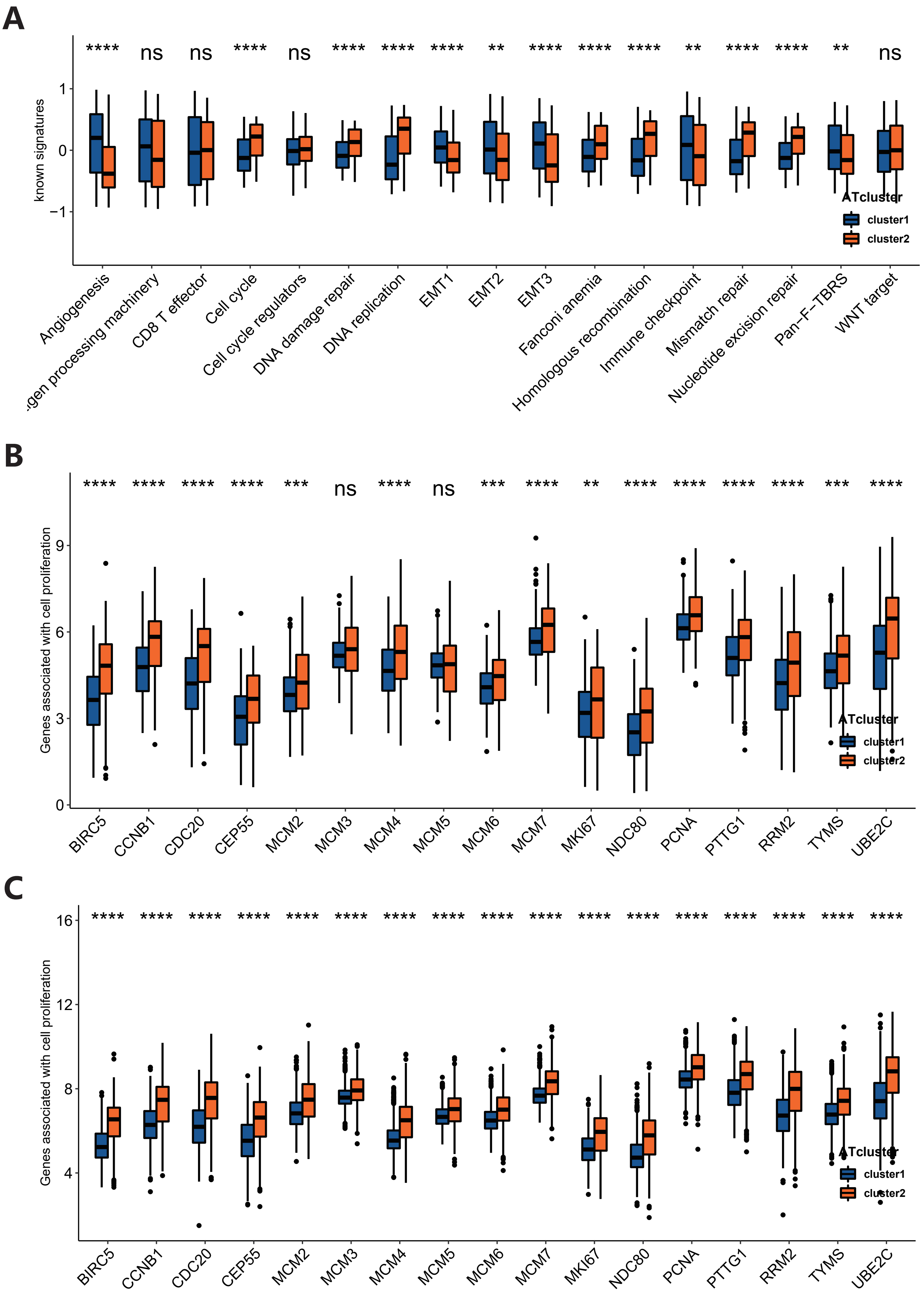

Figure S5: The two autophagy clusters had different well-known signature patterns in TCGA cohort (A), and proliferation-related genes in the meta-cohort (B) and the TCGA cohort (C).

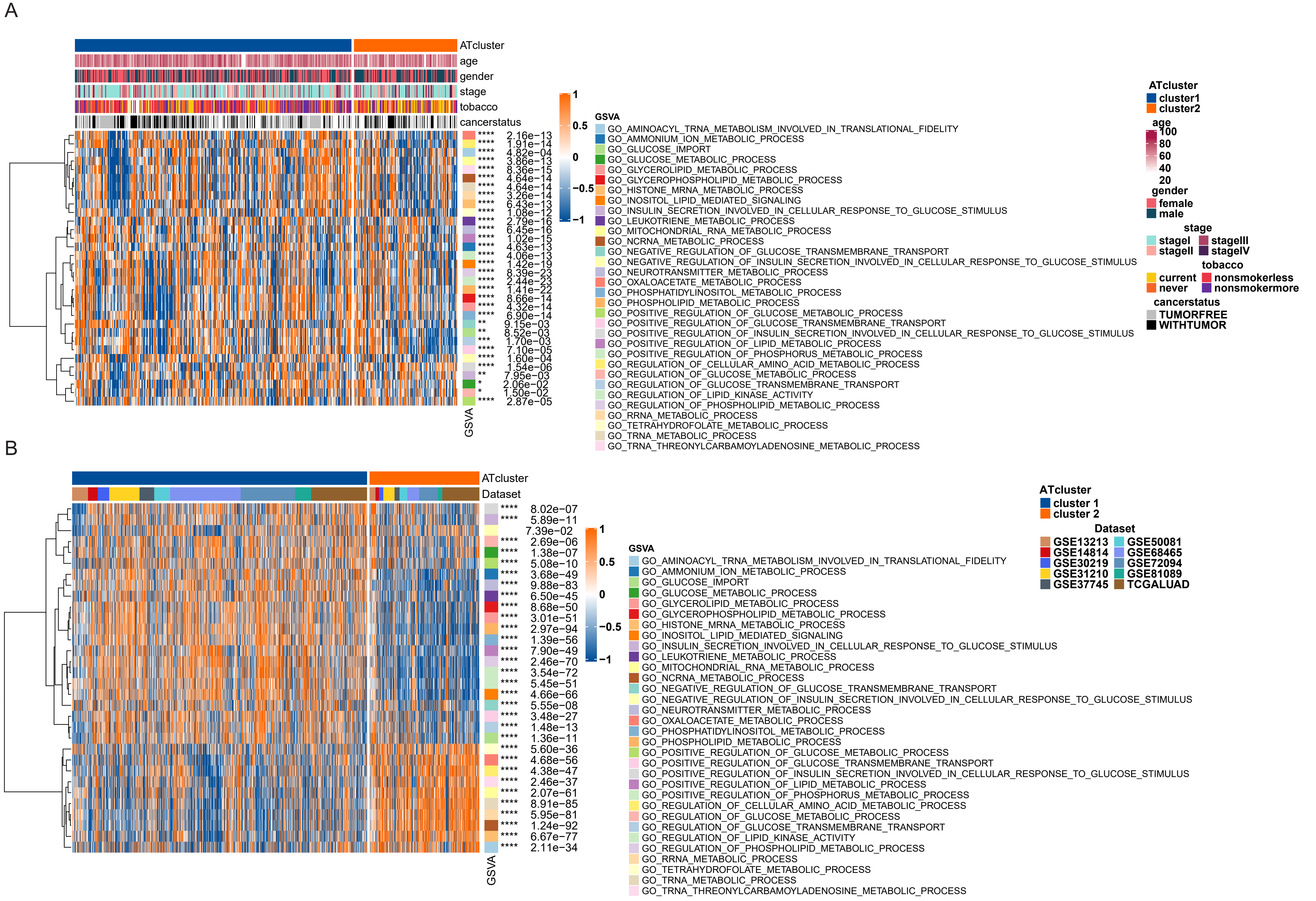

Figure S6: Unsupervised clustering assays showed that metabolic functions were enriched in TCGA cohort (A) and the meta-cohort (B).

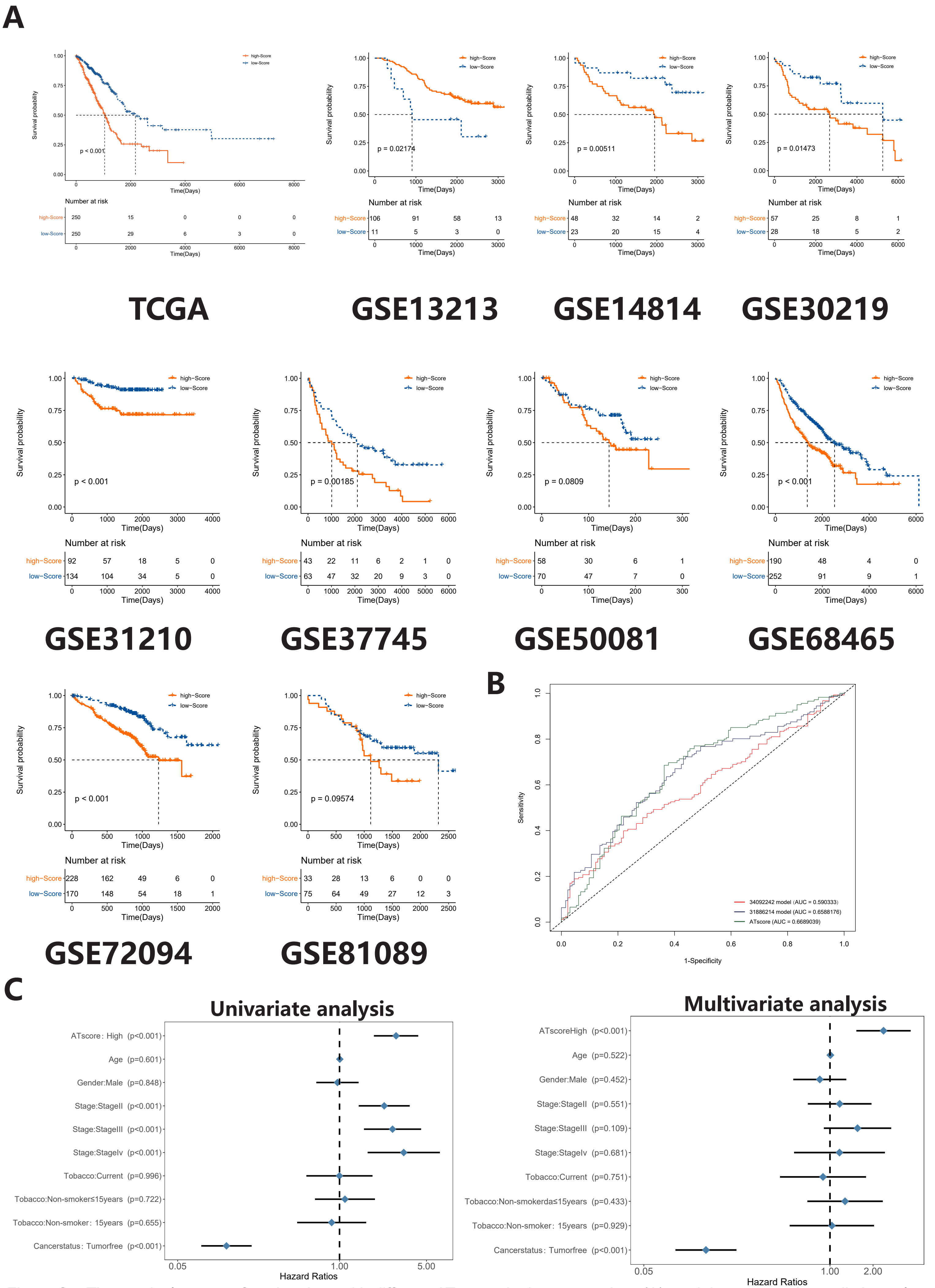

Figure S7: The survival curves of each cohort with different ATscores in the meta-cohort (A), and the prognostic predictive value of ATscore were validated in most datasets . The ROC curve showed that ATscore can be a sensitive marker for survival of LUAD patients, and the AUC of ATscore was bigger than other reported models (B). The COX regression analyses in TCGA cohort showed that ATscore can be an independent risk fator for LUAD patients (C).

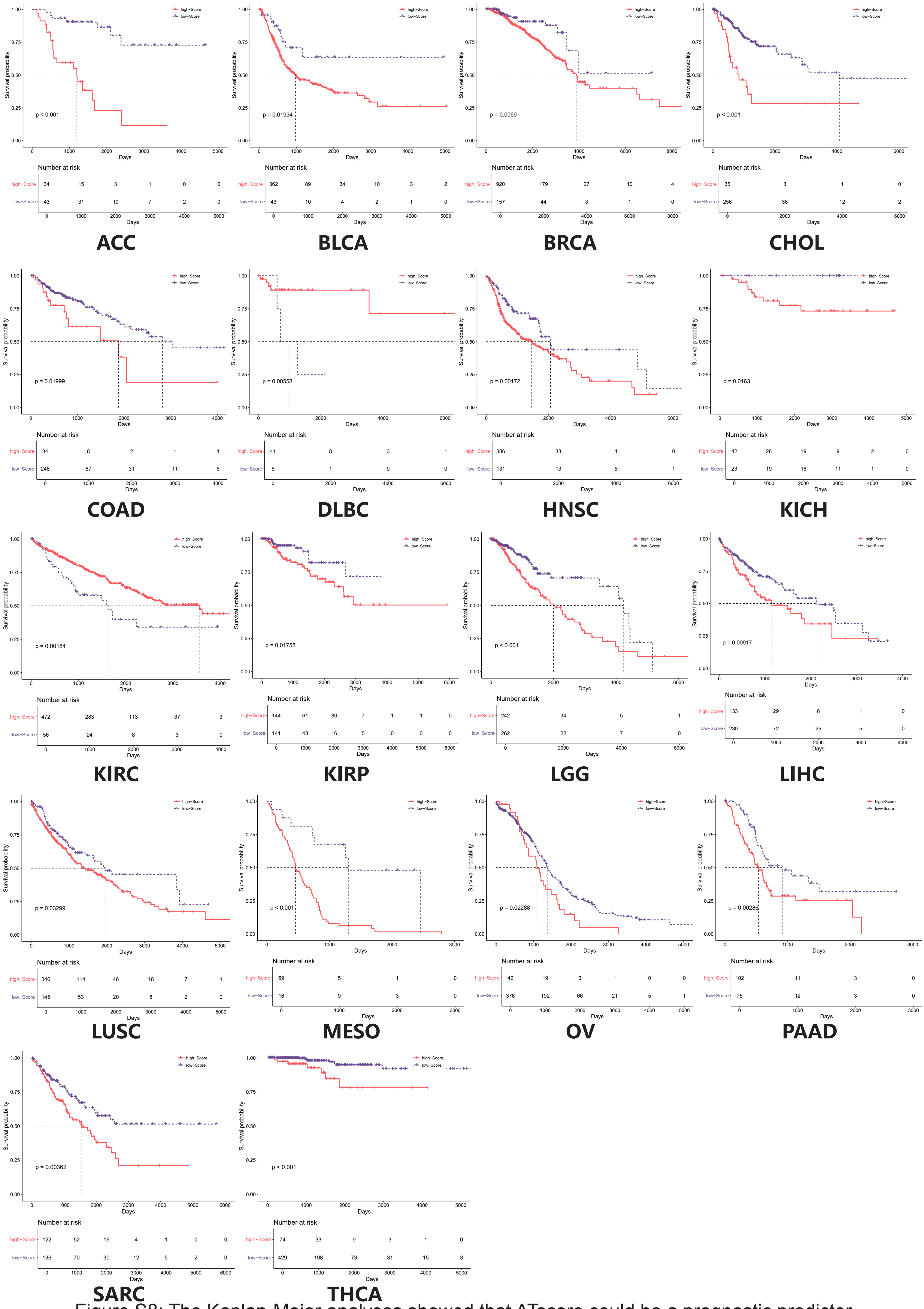

Figure S8: The Kaplan-Meier analyses showed that ATscore could be a prognostic predictor for multiple cancer diseases in TCGA cohort.

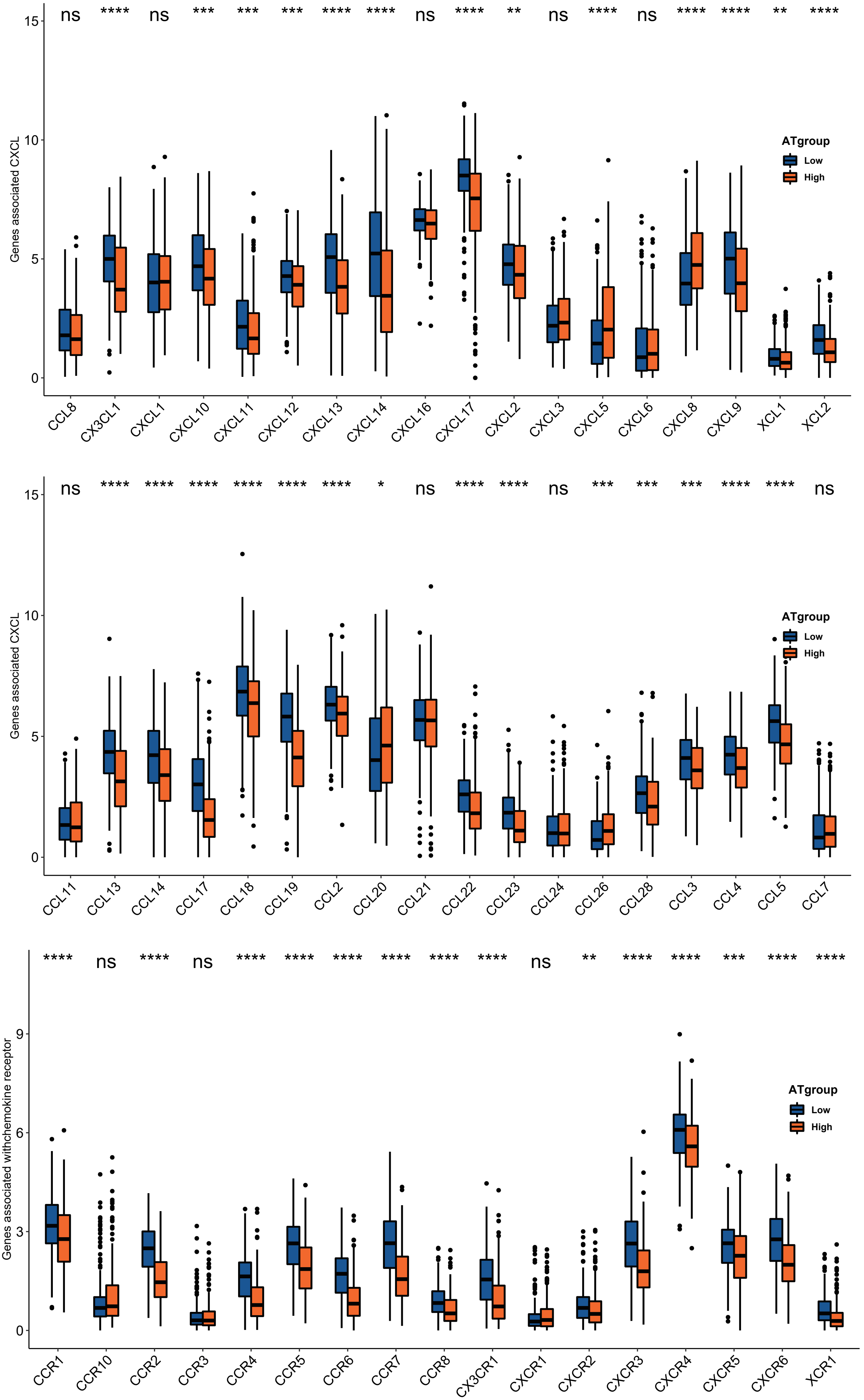

Figure S9: The patients from TCGA cohort with various ATscore had different expression levels of chemokines and receptors . *P＜0.05, **P＜0.01, ***P＜0.001, ****P＜0.0001.

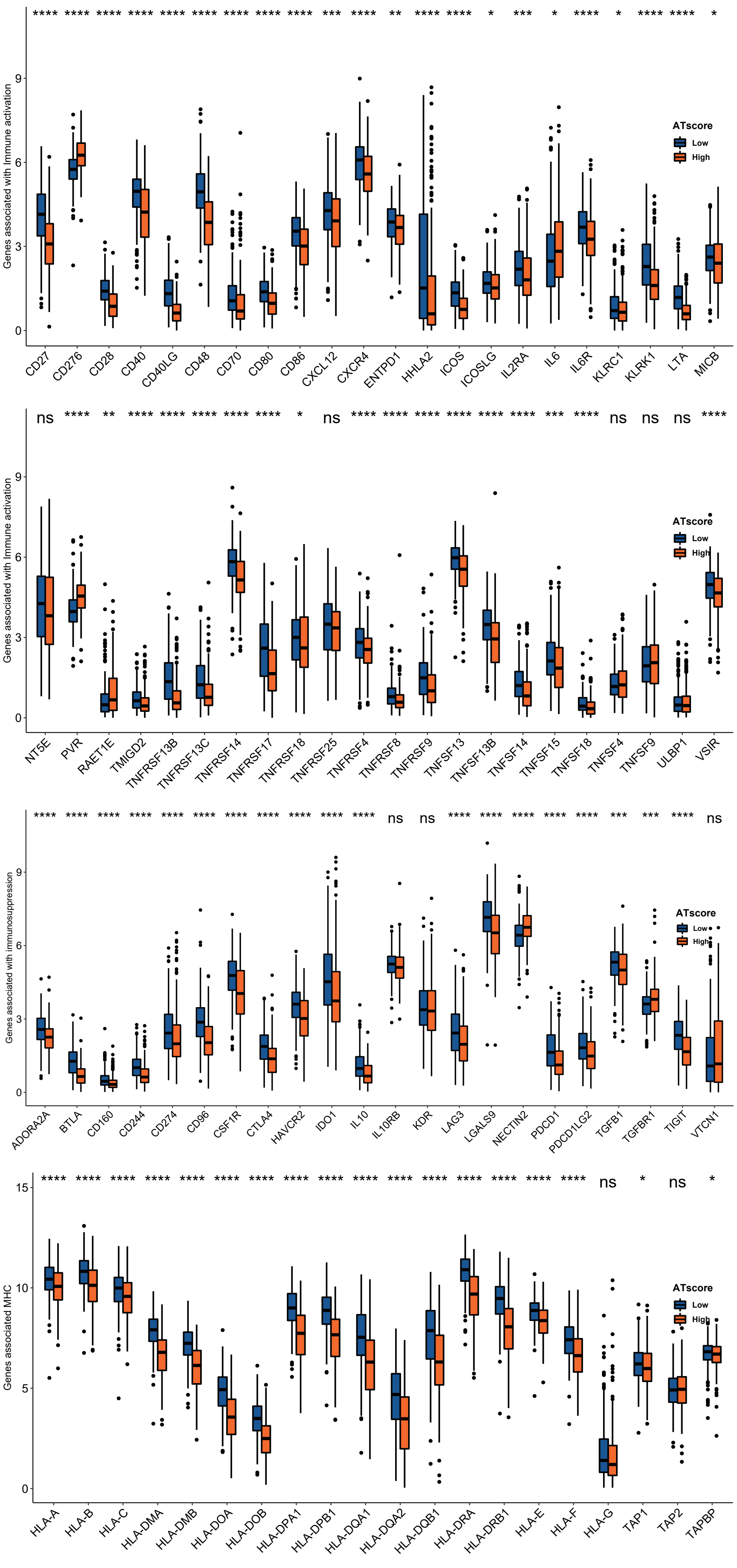

Figure S10: The patients from TCGA cohort with various ATscore had different expression levels of immunosuppression genes, immunoactivation genes and MHC. *P＜0.05, **P＜0.01, ***P＜0.001, ****P＜0.0001.

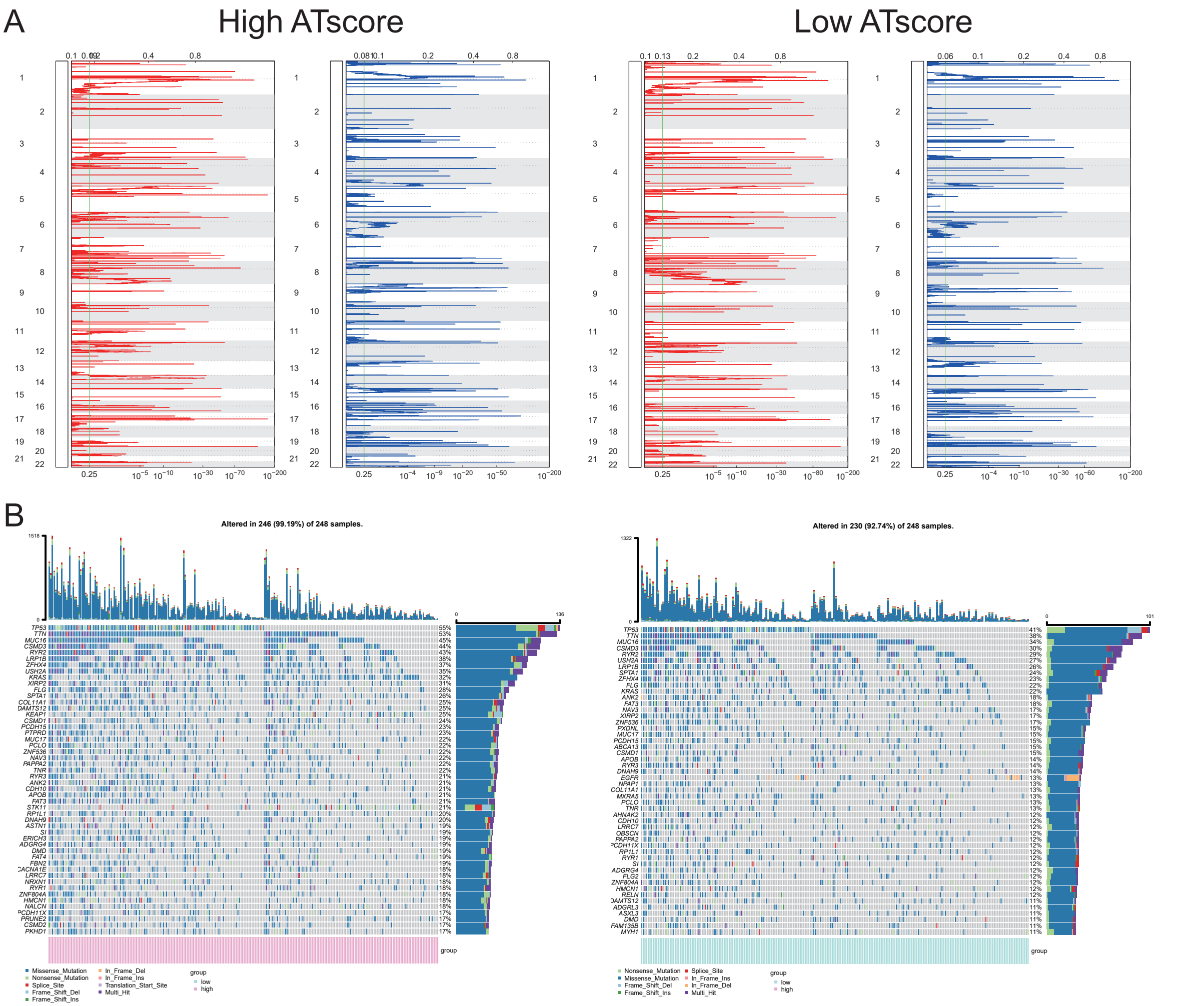

Figure S11: The distinct genomic profiles in LUAD patients from TCGA cohort with various ATscore levels. the results of CNA analysis revealed that LUAD patients from TCGA cohort with different ATscores had various copy number alterations (A). The analysis of somatic mutation profiles indicated higher frequency of TP53, TTN, MUC16, CSMD3 and RYR2 mutations in high ATscore group (B).

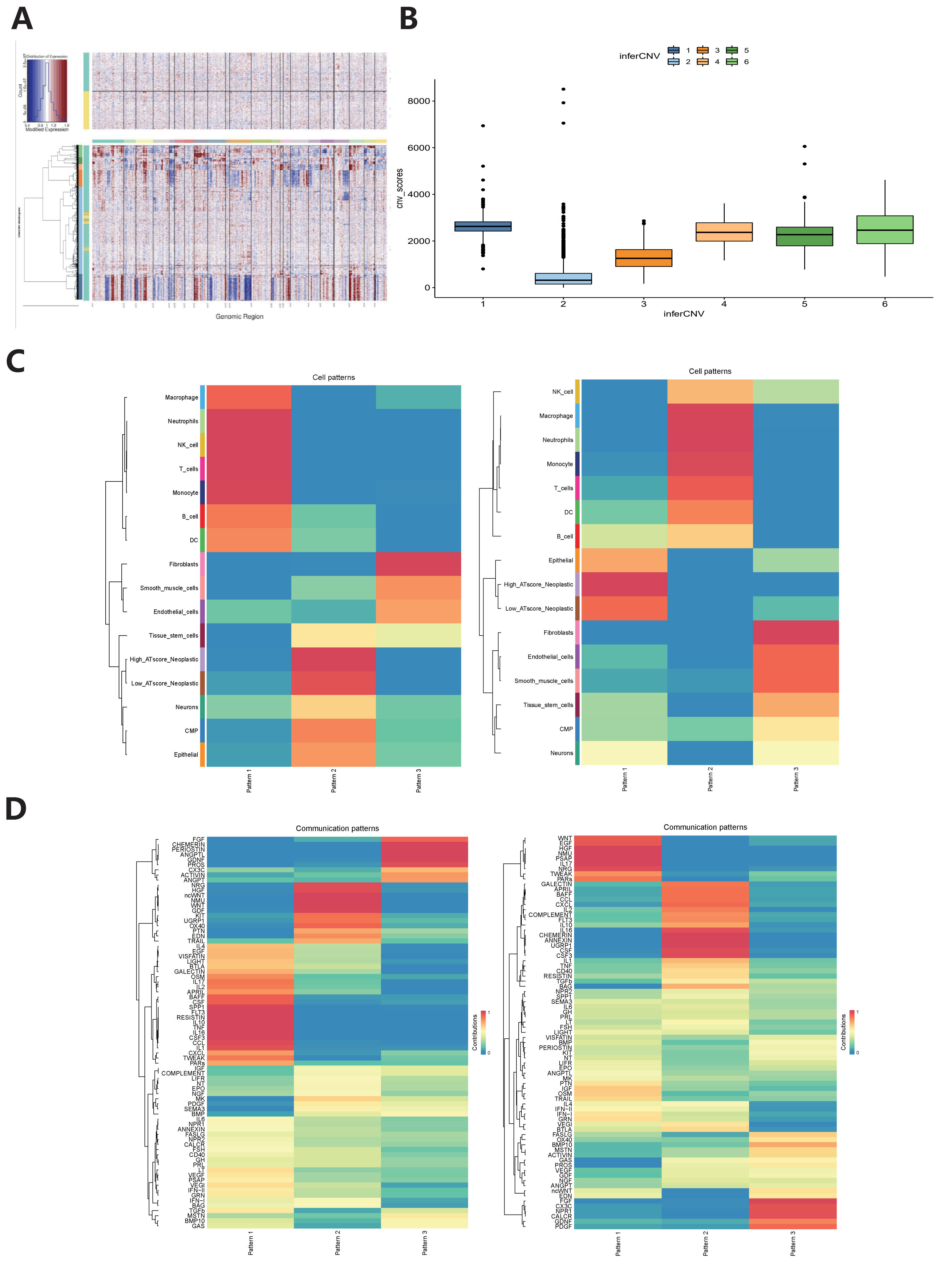

Figure S12: Epithelioid cells of LUAD tumor had different CNV signatures, and the group 2 (wathet blue) had similar CNV signature with the reference (A). The group 2 (wathet blue) had the lowest CNVscores levels (B). The single-cell RNA sequencing data revealed diversity of communication patterns between neoplastic with different ATscores. (C, D).

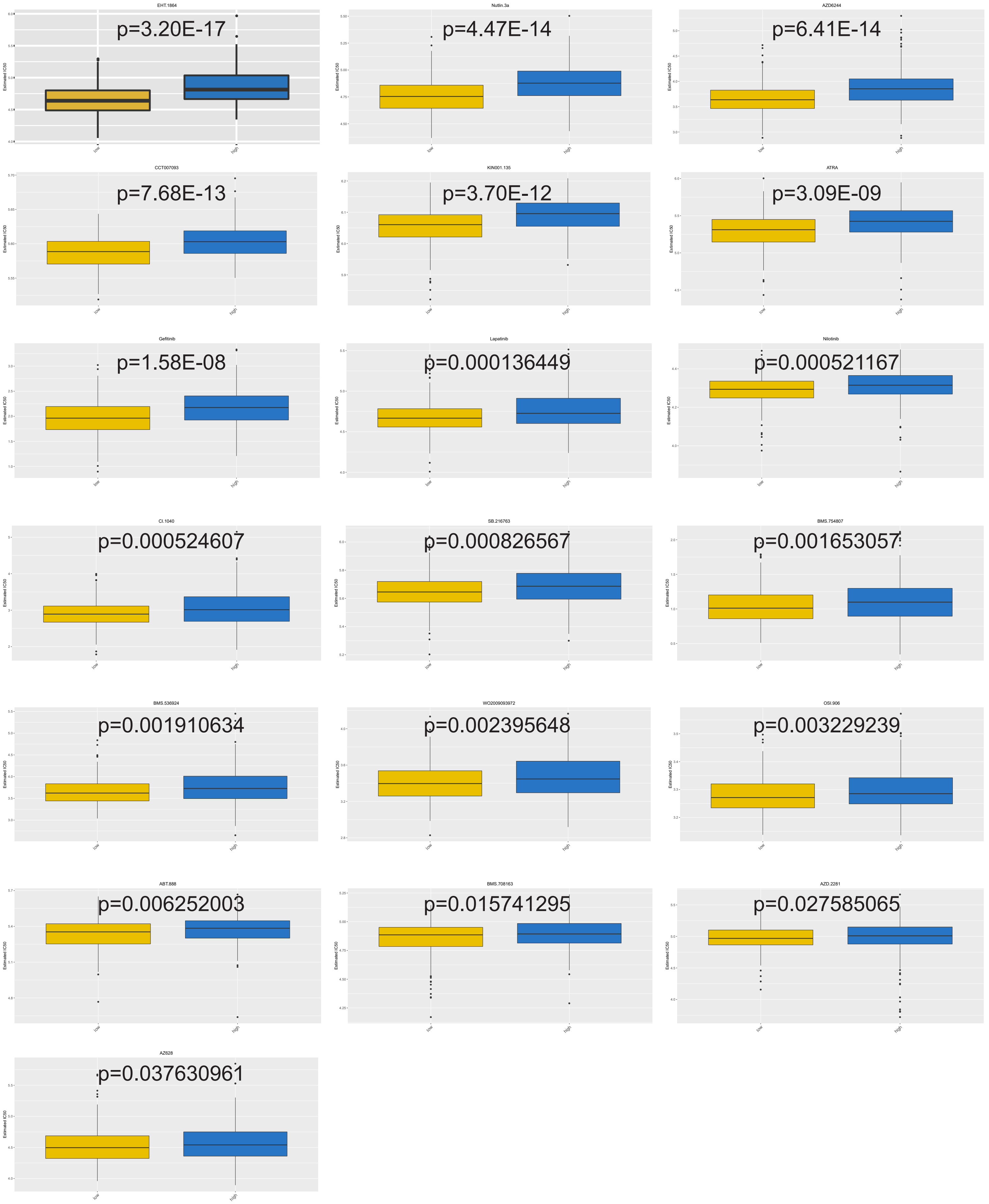

Figure S13: Estimated IC50 values revealed that patients with higher ATscores had significantly higher IC50 value in many chemotherapy molecules compared with those with lower ATscore.
