# Supplementary material for: The Comprehensive Analysis Identified an Autophagy Signature for the Prognosis and the Immunotherapy Efficiency Prediction in Lung Adenocarcinoma
Source: Front Immunol. 2022 Apr 22;13:749241. doi: 10.3389/fimmu.2022.749241 (PMC9072793; doi:10.3389/fimmu.2022.749241)
Supplement: Supplementary file 2 [file DataSheet_2.pdf]

Table S1: 232 autophagy regulators from autophagy database HADb (Human Autophagy I

GeneId	Name
55626	autophagy/beclin-1 regulator 1
8542	apolipoprotein L, 1
405	aryl hydrocarbon receptor nuclear translocator
410	arylsulfatase A
411	arylsulfatase B
468	activating transcription factor 4 (tax-responsive enhancer element B67)
22926	activating transcription factor 6
83734	ATG10 autophagy related 10 homolog (S. cerevisiae)
9140	ATG12 autophagy related 12 homolog (S. cerevisiae)
55054	ATG16 autophagy related 16-like 1 (S. cerevisiae)
89849	ATG16 autophagy related 16-like 2 (S. cerevisiae)
23130	ATG2 autophagy related 2 homolog A (S. cerevisiae)
55102	ATG2 autophagy related 2 homolog B (S. cerevisiae)
64422	ATG3 autophagy related 3 homolog (S. cerevisiae)
115201	ATG4 autophagy related 4 homolog A (S. cerevisiae)
23192	ATG4 autophagy related 4 homolog B (S. cerevisiae)
84938	ATG4 autophagy related 4 homolog C (S. cerevisiae)
84971	ATG4 autophagy related 4 homolog D (S. cerevisiae)
9474	ATG5 autophagy related 5 homolog (S. cerevisiae)
10533	ATG7 autophagy related 7 homolog (S. cerevisiae)
79065	ATG9 autophagy related 9 homolog A (S. cerevisiae)
285973	ATG9 autophagy related 9 homolog B (S. cerevisiae)
471	5-aminoimidazole-4-carboxamide ribonucleotide formyltransferase/IMP cyclohydrolase
573	BCL2-associated athanogene
9531	BCL2-associated athanogene 3
578	BCL2-antagonist/killer 1
581	BCL2-associated X protein
596	B-cell CLL/lymphoma 2
598	BCL2-like 1
8678	beclin 1, autophagy related
637	BH3 interacting domain death agonist
332	baculoviral IAP repeat-containing 5
57448	baculoviral IAP repeat-containing 6
662	BCL2/adenovirus E1B 19kDa interacting protein 1
664	BCL2/adenovirus E1B 19kDa interacting protein 3
665	BCL2/adenovirus E1B 19kDa interacting protein 3-like
60673	chromosome 12 open reading frame 44
23591	chromosome 17 open reading frame 88
10241	calcium binding and coiled-coil domain 2
10645	calcium/calmodulin-dependent protein kinase kinase 2, beta
821	calnexin
823	calpain 1, (mu/I) large subunit
11132	calpain 10
824	calpain 2, (m/II) large subunit
826	calpain, small subunit 1
834	caspase 1, apoptosis-related cysteine peptidase (interleukin 1, beta, convertase)
836	caspase 3, apoptosis-related cysteine peptidase
837	caspase 4, apoptosis-related cysteine peptidase
841	caspase 8, apoptosis-related cysteine peptidase
6347	chemokine (C-C motif) ligand 2
729230	chemokine (C-C motif) receptor 2
4179	CD46 molecule, complement regulatory protein
1026	cyclin-dependent kinase inhibitor 1A (p21, Cip1)
1027	cyclin-dependent kinase inhibitor 1B (p27, Kip1)
1029	cyclin-dependent kinase inhibitor 2A (melanoma, p16, inhibits CDK4)
8837	CASP8 and FADD-like apoptosis regulator

25978	chromatin modifying protein 2B
128866	chromatin modifying protein 4B
1201	ceroid-lipofuscinosis, neuronal 3
1508	cathepsin B
1509	cathepsin D
1514	cathepsin L1
6376	chemokine (C-X3-C motif) ligand 1
7852	chemokine (C-X-C motif) receptor 4
1612	death-associated protein kinase 1
23604	death-associated protein kinase 2
1649	DNA-damage-inducible transcript 3
9077	DIRAS family, GTP-binding RAS-like 3
10395	deleted in liver cancer 1
3337	DnaJ (Hsp40) homolog, subfamily B, member 1
4189	DnaJ (Hsp40) homolog, subfamily B, member 9
55332	DNA-damage regulated autophagy modulator 1
9695	ER degradation enhancer, mannosidase alpha-like 1
1938	eukaryotic translation elongation factor 2
29904	eukaryotic elongation factor-2 kinase
1956	epidermal growth factor receptor (erythroblastic leukemia viral (v-erb-b) oncogene homolog
5610	eukaryotic translation initiation factor 2-alpha kinase 2
9451	eukaryotic translation initiation factor 2-alpha kinase 3
1965	eukaryotic translation initiation factor 2, subunit 1 alpha, 35kDa
1978	eukaryotic translation initiation factor 4E binding protein 1
1981	eukaryotic translation initiation factor 4 gamma, 1
2064	v-erb-b2 erythroblastic leukemia viral oncogene homolog 2, neuro/glioblastoma derived on
2081	endoplasmic reticulum to nucleus signaling 1
30001	ERO1-like (S. cerevisiae)
8772	Fas (TNFRSF6)-associated via death domain
55578	family with sequence similarity 48, member A
355	Fas (TNF receptor superfamily, member 6)
2280	FK506 binding protein 1A, 12kDa
2281	FK506 binding protein 1B, 12.6 kDa
2353	FBJ murine osteosarcoma viral oncogene homolog
2308	forkhead box O1
2309	forkhead box O3
2548	glucosidase, alpha; acid
11337	GABA(A) receptor-associated protein
23710	GABA(A) receptor-associated protein like 1
11345	GABA(A) receptor-associated protein-like 2
2597	glyceraldehyde-3-phosphate dehydrogenase
2773	guanine nucleotide binding protein (G protein), alpha inhibiting activity polypeptide 3
10399	guanine nucleotide binding protein (G protein), beta polypeptide 2-like 1
57120	golgi-associated PDZ and coiled-coil motif containing
2894	glutamate receptor, ionotropic, delta 1
2895	glutamate receptor, ionotropic, delta 2
3065	histone deacetylase 1
10013	histone deacetylase 6
9146	hepatocyte growth factor-regulated tyrosine kinase substrate
3091	hypoxia inducible factor 1, alpha subunit (basic helix-loop-helix transcription factor)
3326	heat shock protein 90kDa alpha (cytosolic), class B member 1
3309	heat shock 70kDa protein 5 (glucose-regulated protein, 78kDa)
3312	heat shock 70kDa protein 8
26353	heat shock 22kDa protein 8
3458	interferon, gamma
3551	inhibitor of kappa light polypeptide gene enhancer in B-cells, kinase beta
9641	inhibitor of kappa light polypeptide gene enhancer in B-cells, kinase epsilon
11009	interleukin 24

345611	immunity-related GTPase family, M
3675	integrin, alpha 3 (antigen CD49C, alpha 3 subunit of VLA-3 receptor)
3655	integrin, alpha 6
3688	integrin, beta 1 (fibronectin receptor, beta polypeptide, antigen CD29 includes MDF2, MSK1[illegible]
3691	integrin, beta 4
3708	inositol 1,4,5-triphosphate receptor, type 1
2548	glucosidase, alpha; acid
11337	GABA(A) receptor-associated protein
23710	GABA(A) receptor-associated protein like 1
11345	GABA(A) receptor-associated protein-like 2
2597	glyceraldehyde-3-phosphate dehydrogenase
2773	guanine nucleotide binding protein (G protein), alpha inhibiting activity polypeptide 3
10399	guanine nucleotide binding protein (G protein), beta polypeptide 2-like 1
57120	golgi-associated PDZ and coiled-coil motif containing
2894	glutamate receptor, ionotropic, delta 1
2895	glutamate receptor, ionotropic, delta 2
9711	KIAA0226
9776	KIAA0652
22863	KIAA0831
3799	kinesin family member 5B
54800	kelch-like 24 (Drosophila)
3916	lysosomal-associated membrane protein 1
3920	lysosomal-associated membrane protein 2
84557	microtubule-associated protein 1 light chain 3 alpha
81631	microtubule-associated protein 1 light chain 3 beta
440738	microtubule-associated protein 1 light chain 3 gamma
5609	mitogen-activated protein kinase kinase 7
5594	mitogen-activated protein kinase 1
5595	mitogen-activated protein kinase 3
5599	mitogen-activated protein kinase 8
9479	mitogen-activated protein kinase 8 interacting protein 1
5601	mitogen-activated protein kinase 9
51360	membrane-bound transcription factor peptidase, site 2
64223	MTOR associated protein, LST8 homolog (S. cerevisiae)
64419	myotubularin related protein 14
2475	mechanistic target of rapamycin (serine/threonine kinase)
4609	v-myc myelocytomatosis viral oncogene homolog (avian)
92345	nuclear assembly factor 1 homolog (S. cerevisiae)
10135	nicotinamide phosphoribosyltransferase
4077	neighbor of BRCA1 gene 1
10787	NCK-associated protein 1
4780	nuclear factor (erythroid-derived 2)-like 2
4790	nuclear factor of kappa light polypeptide gene enhancer in B-cells 1
159296	NK2 transcription factor related, locus 3 (Drosophila)
58484	NLR family, CARD domain containing 4
4864	Niemann-Pick disease, type C1
3084	neuregulin 1
9542	neuregulin 2
10718	neuregulin 3
5034	prolyl 4-hydroxylase, beta polypeptide
5071	Parkinson disease (autosomal recessive, juvenile) 2, parkin
142	poly (ADP-ribose) polymerase 1
8682	phosphoprotein enriched in astrocytes 15
27043	proline, glutamate and leucine rich protein 1
5195	peroxisomal biogenesis factor 14
8504	peroxisomal biogenesis factor 3
5289	phosphoinositide-3-kinase, class 3
30849	phosphoinositide-3-kinase, regulatory subunit 4

65018	PTEN induced putative kinase 1
23645	protein phosphatase 1, regulatory (inhibitor) subunit 15A
5564	protein kinase, AMP-activated, beta 1 non-catalytic subunit
5573	protein kinase, cAMP-dependent, regulatory, type I, alpha (tissue specific extinguisher 1)
5580	protein kinase C, delta
5588	protein kinase C, theta
5728	phosphatase and tensin homolog
5753	PTK6 protein tyrosine kinase 6
8766	RAB11A, member RAS oncogene family
5861	RAB1A, member RAS oncogene family
53917	RAB24, member RAS oncogene family
83452	RAB33B, member RAS oncogene family
5868	RAB5A, member RAS oncogene family
7879	RAB7A, member RAS oncogene family
5879	ras-related C3 botulinum toxin substrate 1 (rho family, small GTP binding protein Rac1)
5894	v-raf-1 murine leukemia viral oncogene homolog 1
5925	retinoblastoma 1
9821	RB1-inducible coiled-coil 1
5970	v-rel reticuloendotheliosis viral oncogene homolog A (avian)
10287	regulator of G-protein signaling 19
6009	Ras homolog enriched in brain
6198	ribosomal protein S6 kinase, 70kDa, polypeptide 1
57521	regulatory associated protein of MTOR, complex 1
56681	SAR1 homolog A (S. cerevisiae)
5265	serpin peptidase inhibitor, clade A (alpha-1 antiproteinase, antitrypsin), member 1
83667	sestrin 2
51100	SH3-domain GRB2-like endophilin B1
23411	sirtuin (silent mating type information regulation 2 homolog) 1 (S. cerevisiae)
22933	sirtuin (silent mating type information regulation 2 homolog) 2 (S. cerevisiae)
8877	sphingosine kinase 1
83985	spinster homolog 1 (Drosophila)
8878	sequestosome 1
6767	suppression of tumorigenicity 13 (colon carcinoma) (Hsp70 interacting protein)
6794	serine/threonine kinase 11
29110	TANK-binding kinase 1
10548	transmembrane 9 superfamily member 1
81671	transmembrane protein 49
157753	transmembrane protein 74
8743	tumor necrosis factor (ligand) superfamily, member 10
7157	tumor protein p53
58476	tumor protein p53 inducible nuclear protein 2
8626	tumor protein p63
7161	tumor protein p73
7248	tuberous sclerosis 1
7249	tuberous sclerosis 2
286319	tumor suppressor candidate 1
8408	unc-51-like kinase 1 (C. elegans)
9706	unc-51-like kinase 2 (C. elegans)
25989	unc-51-like kinase 3 (C. elegans)
9100	ubiquitin specific peptidase 10
7405	UV radiation resistance associated gene
9341	vesicle-associated membrane protein 3 (cellubrevin)
6845	vesicle-associated membrane protein 7
7422	vascular endothelial growth factor A
23001	WD repeat and FYVE domain containing 3
11152	WD repeat domain 45
56270	WDR45-like
55062	WD repeat domain, phosphoinositide interacting 1

26100 WD repeat domain, phosphoinositide interacting 2
53349 zinc finger, FYVE domain containing 1

Database)
Symbol
AMBRA1
APOL1
ARNT
ARSA
ARSB
ATF4
ATF6
ATG10
ATG12
ATG16L1
ATG16L2
ATG2A
ATG2B
ATG3
ATG4A
ATG4B
ATG4C
ATG4D
ATG5
ATG7
ATG9A
ATG9B
ATIC
BAG1
BAG3
BAK1
BAX
BCL2
BCL2L1
BECN1
BID
BIRC5
BIRC6
BNIP1
BNIP3
BNIP3L
C12orf44
C17orf88
CALCOCO2
CAMKK2
CANX
CAPN1
CAPN10
CAPN2
CAPNS1
CASP1
CASP3
CASP4
CASP8
CCL2
CCR2
CD46
CDKN1A
CDKN1B
CDKN2A
CFLAR

CHMP2B
CHMP4B
CLN3
CTSB
CTSD
CTSL1
CX3CL1
CXCR4
DAPK1
DAPK2
DDIT3
DIRAS3
DLC1
DNAJB1
DNAJB9
DRAM1
EDEM1
EEF2
EEF2K
EGFR
EIF2AK2
EIF2AK3
EIF2S1
EIF4EBP1
EIF4G1
ERBB2
ERN1
ERO1L
FADD
FAM48A
FAS
FKBP1A
FKBP1B
FOS
FOXO1
FOXO3
GAA
GABARAP
GABARAPL1
GABARAPL2
GAPDH
GNAI3
GNB2L1
GOPC
GRID1
GRID2
HDAC1
HDAC6
HGS
HIF1A
HSP90AB1
HSPA5
HSPA8
HSPB8
IFNG
IKBKB
IKBKE
IL24

IRGM
ITGA3
ITGA6
ITGB1
ITGB4
ITPR1
GAA
GABARAP
GABARAPL1
GABARAPL2
GAPDH
GNAI3
GNB2L1
GOPC
GRID1
GRID2
KIAA0226
KIAA0652
KIAA0831
KIF5B
KLHL24
LAMP1
LAMP2
MAP1LC3A
MAP1LC3B
MAP1LC3C
MAP2K7
MAPK1
MAPK3
MAPK8
MAPK8IP1
MAPK9
MBTPS2
MLST8
MTMR14
MTOR
MYC
NAF1
NAMPT
NBR1
NCKAP1
NFE2L2
NFKB1
NKX2-3
NLRC4
NPC1
NRG1
NRG2
NRG3
P4HB
PARK2
PARP1
PEA15
PELP1
PEX14
PEX3
PIK3C3
PIK3R4

PINK1
PPP1R15A
PRKAB1
PRKAR1A
PRKCD
PRKCQ
PTEN
PTK6
RAB11A
RAB1A
RAB24
RAB33B
RAB5A
RAB7A
RAC1
RAF1
RB1
RB1CC1
RELA
RGS19
RHEB
RPS6KB1
RPTOR
SAR1A
SERPINA1
SESN2
SH3GLB1
SIRT1
SIRT2
SPHK1
SPNS1
SQSTM1
ST13
STK11
TBK1
TM9SF1
TMEM49
TMEM74
TNFSF10
TP53
TP53INP2
TP63
TP73
TSC1
TSC2
TUSC1
ULK1
ULK2
ULK3
USP10
UVRAG
VAMP3
VAMP7
VEGFA
WDFY3
WDR45
WDR45L
WIPI1

WIPI2
ZFYVE1

Table S2: Kaplan-Meier analyses of all 232 autophagy regulators in meta-cohort

	characteris	Hazard.Rat	CI95	pvalue	HR(95%CI)
1	AMBRA1	1.016	0.838-1.23	0.870544	1.016(0.838-1.231)
2	APOL1	1.232	1.145-1.32	2.55E-08	1.232(1.145-1.326)
3	ARNT	0.935	0.804-1.08	0.377108	0.935(0.804-1.086)
4	ARSA	0.897	0.799-1.00	0.066201	0.897(0.799-1.007)
5	ARSB	1.113	0.947-1.30	0.193392	1.113(0.947-1.307)
6	ATF4	1.043	0.89-1.222	0.605996	1.043(0.89-1.222)
7	ATF6	0.914	0.783-1.06	0.2532	0.914(0.783-1.067)
8	ATG10	1.06	0.892-1.26	0.507522	1.06(0.892-1.26)
9	ATG16L1	1.126	0.952-1.33	0.16628	1.126(0.952-1.332)
10	ATG2A	0.848	0.739-0.97	0.018849	0.848(0.739-0.973)
11	ATG2B	0.793	0.691-0.91	0.001053	0.793(0.691-0.911)
12	ATG3	1.252	1.042-1.50	0.016184	1.252(1.042-1.503)
13	ATG4A	0.871	0.74-1.026	0.098278	0.871(0.74-1.026)
14	ATG4B	1.114	0.968-1.28	0.131217	1.114(0.968-1.283)
15	ATG5	1.153	1.011-1.31	0.033865	1.153(1.011-1.315)
16	ATG7	1.38	1.161-1.64	0.00026	1.38(1.161-1.64)
17	ATG9A	1.295	1.102-1.52	0.00169	1.295(1.102-1.521)
18	ATIC	1.363	1.196-1.55	3.49E-06	1.363(1.196-1.554)
19	BAG1	0.943	0.846-1.05	0.285899	0.943(0.846-1.051)
20	BAG3	1.166	1.052-1.29	0.003519	1.166(1.052-1.293)
21	BAK1	1.297	1.129-1.49	0.00024	1.297(1.129-1.49)
22	BAX	1.103	0.966-1.26	0.148243	1.103(0.966-1.26)
23	BCL2	0.789	0.686-0.90	0.000871	0.789(0.686-0.907)
24	BCL2L1	1.196	1.06-1.349	0.00367	1.196(1.06-1.349)
25	BID	1.285	1.16-1.425	1.69E-06	1.285(1.16-1.425)
26	BIRC5	1.276	1.196-1.36	1.82E-13	1.276(1.196-1.361)
27	BNIP1	1.114	0.945-1.31	0.199417	1.114(0.945-1.313)
28	BNIP3	1.05	0.971-1.13	0.222256	1.05(0.971-1.136)
29	BNIP3L	0.882	0.782-0.99	0.038024	0.882(0.782-0.993)
30	CALCOCO	0.781	0.683-0.89	0.000294	0.781(0.683-0.893)
31	CAMKK2	1.34	1.135-1.58	0.00053	1.34(1.135-1.581)
32	CANX	1.058	0.943-1.18	0.335964	1.058(0.943-1.188)
33	CAPN1	1.122	0.987-1.27	0.079423	1.122(0.987-1.276)
34	CAPN10	0.933	0.812-1.07	0.32202	0.933(0.812-1.071)
35	CAPN2	0.903	0.803-1.01	0.090079	0.903(0.803-1.016)
36	CAPNS1	1.202	1.044-1.38	0.010686	1.202(1.044-1.384)
37	CASP1	0.945	0.87-1.027	0.184249	0.945(0.87-1.027)
38	CASP3	1.155	1.001-1.33	0.04909	1.155(1.001-1.333)
39	CASP4	1.136	1.007-1.28	0.037416	1.136(1.007-1.281)
40	CASP8	1.083	0.95-1.234	0.231303	1.083(0.95-1.234)
41	CCL2	1.009	0.952-1.07	0.752382	1.009(0.952-1.07)
42	CCR2	0.74	0.668-0.81	5.35E-09	0.74(0.668-0.818)
43	CD46	0.942	0.856-1.03	0.216149	0.942(0.856-1.036)
44	CDKN1A	1.249	1.136-1.37	4.11E-06	1.249(1.136-1.372)
45	CDKN1B	0.879	0.779-0.99	0.035385	0.879(0.779-0.991)
46	CDKN2A	1.057	1.009-1.10	0.01856	1.057(1.009-1.107)
47	CFLAR	0.898	0.804-1.00	0.056158	0.898(0.804-1.003)
48	CHMP2B	1.095	0.973-1.23	0.133155	1.095(0.973-1.234)
49	CLN3	0.841	0.741-0.95	0.007335	0.841(0.741-0.954)
50	CTSB	1.178	1.06-1.309	0.002258	1.178(1.06-1.309)
51	CTSD	0.926	0.847-1.01	0.094151	0.926(0.847-1.013)
52	CX3CL1	0.909	0.86-0.962	0.000977	0.909(0.86-0.962)
53	CXCR4	0.944	0.871-1.02	0.15839	0.944(0.871-1.023)
54	DAPK1	0.792	0.717-0.87	4.25E-06	0.792(0.717-0.875)
55	DAPK2	0.718	0.653-0.79	1.43E-11	0.718(0.653-0.791)
56	DDIT3	1.029	0.936-1.13	0.555745	1.029(0.936-1.131)
57	DIRAS3	0.951	0.894-1.01	0.116315	0.951(0.894-1.012)

58	DLC1	0.833	0.772-0.9	3.45E-06	0.833(0.772-0.9)
59	DNAJB1	1.083	0.992-1.18	0.074456	1.083(0.992-1.181)
60	DNAJB9	0.859	0.775-0.95	0.00361	0.859(0.775-0.951)
61	DRAM1	0.818	0.763-0.87	9.49E-09	0.818(0.763-0.876)
62	EDEM1	0.824	0.737-0.92	0.000635	0.824(0.737-0.921)
63	EEF2	0.8	0.69-0.929	0.003381	0.8(0.69-0.929)
64	EGFR	1.005	0.933-1.08	0.886507	1.005(0.933-1.084)
65	EIF2AK2	1.202	1.059-1.36	0.004398	1.202(1.059-1.363)
66	EIF2AK3	0.835	0.739-0.94	0.00375	0.835(0.739-0.943)
67	EIF2S1	1.197	1.06-1.351	0.003619	1.197(1.06-1.351)
68	EIF4EBP1	1.182	1.102-1.26	3.14E-06	1.182(1.102-1.268)
69	EIF4G1	1.239	1.092-1.40	0.000889	1.239(1.092-1.406)
70	ERBB2	0.949	0.862-1.04	0.284249	0.949(0.862-1.045)
71	ERN1	0.895	0.793-1.01	0.070954	0.895(0.793-1.01)
72	FADD	1.358	1.192-1.54	3.98E-06	1.358(1.192-1.546)
73	FAS	0.953	0.876-1.03	0.253205	0.953(0.876-1.035)
74	FKBP1A	1.29	1.131-1.47	0.000143	1.29(1.131-1.471)
75	FKBP1B	1.016	0.934-1.10	0.705443	1.016(0.934-1.106)
76	FOS	0.94	0.892-0.99	0.023359	0.94(0.892-0.992)
77	FOXO1	0.919	0.826-1.02	0.12519	0.919(0.826-1.024)
78	FOXO3	0.958	0.847-1.08	0.494663	0.958(0.847-1.084)
79	GAA	1.028	0.93-1.136	0.587764	1.028(0.93-1.136)
80	GABARAP	0.881	0.733-1.05	0.176006	0.881(0.733-1.058)
81	GABARAPL	1.052	0.951-1.16	0.324884	1.052(0.951-1.163)
82	GABARAPL	0.913	0.788-1.05	0.223825	0.913(0.788-1.057)
83	GAPDH	1.621	1.448-1.81	3.68E-17	1.621(1.448-1.813)
84	GNAI3	1.384	1.203-1.59	5.60E-06	1.384(1.203-1.592)
85	HDAC1	1.23	1.071-1.41	0.003328	1.23(1.071-1.412)
86	HDAC6	0.922	0.796-1.06	0.27773	0.922(0.796-1.068)
87	HGS	1.159	1.019-1.31	0.025075	1.159(1.019-1.318)
88	HIF1A	1.178	1.055-1.31	0.003434	1.178(1.055-1.314)
89	HSP90AB1	1.024	0.915-1.14	0.679182	1.024(0.915-1.147)
90	HSPA5	1.034	0.906-1.18	0.621113	1.034(0.906-1.18)
91	HSPA8	1.168	1.036-1.31	0.011371	1.168(1.036-1.317)
92	HSPB8	0.845	0.783-0.91	1.27E-05	0.845(0.783-0.911)
93	IFNG	1.031	0.955-1.11	0.434815	1.031(0.955-1.112)
94	IKBKB	0.787	0.705-0.87	2.00E-05	0.787(0.705-0.879)
95	IL24	1.021	0.91-1.145	0.728417	1.021(0.91-1.145)
96	ITGA3	1.075	1-1.155	0.050681	1.075(1-1.155)
97	ITGA6	1.203	1.119-1.29	5.84E-07	1.203(1.119-1.294)
98	ITGB1	1.403	1.23-1.601	4.94E-07	1.403(1.23-1.601)
99	ITGB4	1.251	1.169-1.33	9.30E-11	1.251(1.169-1.339)
100	ITPR1	0.818	0.74-0.903	7.73E-05	0.818(0.74-0.903)
101	KIF5B	1.121	0.981-1.28	0.094115	1.121(0.981-1.282)
102	KLHL24	0.923	0.823-1.03	0.169891	0.923(0.823-1.035)
103	LAMP1	0.972	0.86-1.098	0.644712	0.972(0.86-1.098)
104	LAMP2	1	0.891-1.12	0.995281	1(0.891-1.121)
105	MAP1LC3E	0.945	0.808-1.10	0.475597	0.945(0.808-1.105)
106	MAP1LC3C	0.901	0.827-0.98	0.017446	0.901(0.827-0.982)
107	MAP2K7	0.98	0.836-1.15	0.806105	0.98(0.836-1.15)
108	MAPK1	1.139	1.022-1.27	0.019185	1.139(1.022-1.271)
109	MAPK3	0.98	0.842-1.14	0.795344	0.98(0.842-1.141)
110	MAPK8	1.066	0.928-1.22	0.367424	1.066(0.928-1.225)
111	MAPK8IP1	0.913	0.804-1.03	0.159063	0.913(0.804-1.036)
112	MAPK9	0.928	0.814-1.05	0.265647	0.928(0.814-1.058)
113	MBTPS2	1.305	1.097-1.55	0.002694	1.305(1.097-1.554)
114	MLST8	1.018	0.877-1.18	0.812677	1.018(0.877-1.182)
115	MTMR14	0.988	0.816-1.19	0.902709	0.988(0.816-1.197)
116	MTOR	0.952	0.796-1.13	0.590523	0.952(0.796-1.138)

117	MYC	1.112	1.041-1.18	0.00169	1.112(1.041-1.188)
118	NAMPT	1.064	1.001-1.13	0.044633	1.064(1.001-1.131)
119	NBR1	1.049	0.905-1.21	0.524248	1.049(0.905-1.218)
120	NCKAP1	1.168	1.013-1.34	0.032044	1.168(1.013-1.347)
121	NFE2L2	0.982	0.861-1.12	0.786335	0.982(0.861-1.12)
122	NFKB1	0.929	0.807-1.06	0.302367	0.929(0.807-1.069)
123	NPC1	0.922	0.816-1.04	0.195101	0.922(0.816-1.042)
124	NRG1	1.029	0.921-1.15	0.615086	1.029(0.921-1.15)
125	NRG2	0.93	0.76-1.138	0.480048	0.93(0.76-1.138)
126	P4HB	1.216	1.06-1.394	0.005119	1.216(1.06-1.394)
127	PARP1	1.058	0.919-1.21	0.432852	1.058(0.919-1.218)
128	PEA15	1.127	0.987-1.28	0.077423	1.127(0.987-1.288)
129	PELP1	1.007	0.871-1.16	0.929497	1.007(0.871-1.163)
130	PEX14	0.939	0.797-1.10	0.448887	0.939(0.797-1.106)
131	PEX3	0.935	0.817-1.07	0.334541	0.935(0.817-1.071)
132	PIK3C3	1.006	0.849-1.19	0.947962	1.006(0.849-1.191)
133	PIK3R4	0.908	0.749-1.10	0.3273	0.908(0.749-1.101)
134	PINK1	0.876	0.76-1.009	0.067119	0.876(0.76-1.009)
135	PPP1R15A	0.959	0.873-1.05	0.385139	0.959(0.873-1.054)
136	PRKAB1	0.799	0.713-0.89	9.99E-05	0.799(0.713-0.894)
137	PRKAR1A	0.937	0.832-1.05	0.289218	0.937(0.832-1.056)
138	PRKCD	0.767	0.676-0.87	3.60E-05	0.767(0.676-0.87)
139	PRKCQ	0.885	0.791-0.99	0.032956	0.885(0.791-0.99)
140	PTEN	1.064	0.925-1.22	0.385156	1.064(0.925-1.223)
141	PTK6	1.099	1.023-1.18	0.009779	1.099(1.023-1.18)
142	RAB11A	1.225	1.081-1.38	0.001535	1.225(1.081-1.389)
143	RAB1A	1.166	1.005-1.35	0.043207	1.166(1.005-1.354)
144	RAB33B	0.833	0.716-0.97	0.018391	0.833(0.716-0.97)
145	RAB5A	1.246	1.067-1.45	0.005344	1.246(1.067-1.454)
146	RAB7A	1.379	1.145-1.66	0.000718	1.379(1.145-1.662)
147	RAC1	1.287	1.112-1.49	0.00071	1.287(1.112-1.49)
148	RAF1	0.875	0.735-1.04	0.13437	0.875(0.735-1.042)
149	RB1	1.007	0.88-1.153	0.914911	1.007(0.88-1.153)
150	RB1CC1	0.964	0.857-1.08	0.549679	0.964(0.857-1.086)
151	RELA	1.178	0.997-1.39	0.054502	1.178(0.997-1.393)
152	RGS19	1.022	0.904-1.15	0.73236	1.022(0.904-1.155)
153	RHEB	1.055	0.916-1.21	0.454487	1.055(0.916-1.216)
154	RPS6KB1	1.12	0.969-1.29	0.126343	1.12(0.969-1.294)
155	SAR1A	1.289	1.122-1.48	0.000327	1.289(1.122-1.481)
156	SERPINA1	0.997	0.953-1.04	0.882809	0.997(0.953-1.042)
157	SH3GLB1	1.332	1.165-1.52	2.82E-05	1.332(1.165-1.523)
158	SIRT1	0.8	0.707-0.90	0.000413	0.8(0.707-0.905)
159	SIRT2	0.786	0.669-0.92	0.003505	0.786(0.669-0.924)
160	SPHK1	1.229	1.147-1.31	5.50E-09	1.229(1.147-1.318)
161	SQSTM1	1.01	0.907-1.12	0.850178	1.01(0.907-1.126)
162	ST13	0.96	0.848-1.08	0.51463	0.96(0.848-1.086)
163	STK11	0.899	0.786-1.02	0.119653	0.899(0.786-1.028)
164	TBK1	1.179	1.025-1.35	0.021074	1.179(1.025-1.357)
165	TM9SF1	1.045	0.906-1.20	0.54703	1.045(0.906-1.206)
166	TNFSF10	0.982	0.92-1.048	0.576154	0.982(0.92-1.048)
167	TP53	0.992	0.902-1.09	0.863668	0.992(0.902-1.091)
168	TP63	0.98	0.915-1.05	0.57616	0.98(0.915-1.051)
169	TP73	0.93	0.819-1.05	0.262984	0.93(0.819-1.056)
170	TSC1	0.881	0.763-1.01	0.082816	0.881(0.763-1.017)
171	TSC2	0.872	0.767-0.99	0.036617	0.872(0.767-0.992)
172	ULK1	1.053	0.935-1.18	0.394337	1.053(0.935-1.185)
173	ULK2	0.801	0.7-0.915	0.001139	0.801(0.7-0.915)
174	USP10	1.089	0.934-1.27	0.276065	1.089(0.934-1.271)
175	UVRAG	0.909	0.784-1.05	0.210058	0.909(0.784-1.055)

176	VAMP3	0.999	0.869-1.14	0.98899	0.999(0.869-1.149)
177	VAMP7	0.997	0.884-1.12	0.957272	0.997(0.884-1.124)
178	VEGFA	1.27	1.175-1.37	1.68E-09	1.27(1.175-1.372)
179	WDFY3	0.892	0.781-1.01	0.09229	0.892(0.781-1.019)
180	WDR45	0.817	0.692-0.96	0.017291	0.817(0.692-0.965)
181	WIPI1	1.045	0.925-1.18	0.475725	1.045(0.925-1.181)
182	WIPI2	1.089	0.937-1.26	0.26499	1.089(0.937-1.266)

Table S3: all DEGs between the two Atclusters

	gene	logFC	AveExpr	t	P.Value	adj.P.Val	B
1	TMEM173	-1.26152	5.975408	-15.6594	2.80E-45	5.78E-41	92.0347
2	NAPSB	-1.46972	3.699747	-14.4646	7.06E-40	7.30E-36	79.76305
3	TMEM119	-1.34325	3.746328	-13.9642	1.15E-37	7.93E-34	74.73846
4	CD4	-1.2181	5.211456	-13.7285	1.23E-36	6.38E-33	72.39866
5	MFAP4	-1.79756	5.305178	-13.6635	2.37E-36	9.78E-33	71.75633
6	GAS7	-1.03584	2.514286	-13.595	4.69E-36	1.62E-32	71.08069
7	RASSF2	-1.01074	2.886797	-13.5728	5.86E-36	1.73E-32	70.86229
8	ARHGDIB	-1.04177	7.831379	-13.5463	7.63E-36	1.97E-32	70.60147
9	CD37	-1.34618	5.070262	-13.497	1.25E-35	2.86E-32	70.11799
10	ARHGAP3(	-1.03561	3.356019	-13.3406	5.88E-35	1.01E-31	68.58696
11	PODN	-1.31445	3.95012	-13.3073	8.18E-35	1.30E-31	68.26204
12	SPARCL1	-1.23674	5.816138	-13.084	7.38E-34	9.53E-31	66.09325
13	PTGDS	-1.78414	6.226466	-13.0572	9.59E-34	1.10E-30	65.83456
14	AOC3	-1.33874	4.076053	-12.9605	2.47E-33	2.55E-30	64.90119
15	C7	-2.07836	4.213026	-12.9263	3.45E-33	3.40E-30	64.5722
16	A2M	-1.30979	7.755283	-12.8699	5.97E-33	5.61E-30	64.03067
17	IL16	-1.04519	3.324508	-12.7307	2.31E-32	1.91E-29	62.69922
18	SELPLG	-1.06964	4.189691	-12.6374	5.68E-32	4.12E-29	61.81092
19	HLA.DPB1	-1.45877	8.157953	-12.627	6.28E-32	4.32E-29	61.71209
20	CELF2	-1.08284	3.549113	-12.6225	6.55E-32	4.37E-29	61.66935
21	CIITA	-1.19808	3.31123	-12.518	1.79E-31	1.09E-28	60.67863
22	HLA.DOA	-1.44401	4.1987	-12.5109	1.92E-31	1.13E-28	60.61086
23	SLCO2B1	-1.25718	4.451268	-12.4653	2.97E-31	1.66E-28	60.18066
24	SLC34A2	-2.24987	8.300537	-12.4575	3.20E-31	1.72E-28	60.10682
25	CX3CL1	-1.74811	4.491483	-12.4559	3.25E-31	1.72E-28	60.09155
26	NAPSA	-2.87237	9.296016	-12.4392	3.81E-31	1.97E-28	59.93435
27	SPN	-1.06165	2.703594	-12.4256	4.34E-31	2.19E-28	59.8058
28	SASH3	-1.05699	3.59127	-12.4083	5.12E-31	2.46E-28	59.64259
29	CD74	-1.36868	11.51898	-12.3949	5.82E-31	2.73E-28	59.51652
30	DOCK2	-1.00221	3.133288	-12.378	6.84E-31	3.14E-28	59.35741
31	SYNE1	-1.0985	4.1834	-12.3294	1.09E-30	4.78E-28	58.90135
32	RPS6KA2	-1.08859	4.424941	-12.2847	1.66E-30	6.74E-28	58.48256
33	LSP1	-1.13275	5.606312	-12.275	1.82E-30	7.25E-28	58.39133
34	FRZB	-1.09038	2.524748	-12.2367	2.62E-30	1.02E-27	58.03304
35	JAML	-1.19959	3.791684	-12.2079	3.45E-30	1.32E-27	57.76372
36	FCMR	-1.05209	3.080767	-12.191	4.04E-30	1.52E-27	57.60676
37	PRELP	-1.34266	3.656484	-12.1891	4.11E-30	1.52E-27	57.58875
38	SLIT3	-1.20571	2.310896	-12.1414	6.46E-30	2.30E-27	57.14409
39	PLD4	-1.08378	2.051876	-12.0901	1.05E-29	3.44E-27	56.66722
40	ROS1	-1.77924	3.245294	-12.0839	1.11E-29	3.53E-27	56.60981
41	ITGAL	-1.10794	3.370154	-12.0419	1.65E-29	5.09E-27	56.22018
42	PARVG	-1.0377	3.800194	-12.0296	1.85E-29	5.55E-27	56.10615
43	PAPLN	-1.0004	3.025518	-11.9871	2.76E-29	8.04E-27	55.71344
44	ADGRF5	-1.68047	4.949497	-11.9769	3.04E-29	8.43E-27	55.61925
45	TPSAB1	-1.71118	4.852625	-11.9758	3.07E-29	8.43E-27	55.60889
46	ELN	-1.64615	4.763539	-11.9638	3.43E-29	8.98E-27	55.49818
47	HLA.DMA	-1.20859	7.259471	-11.9578	3.63E-29	9.38E-27	55.44292
48	LTBP2	-1.2608	4.673334	-11.9384	4.36E-29	1.05E-26	55.26302
49	ABI3BP	-1.34114	3.264953	-11.9054	5.93E-29	1.39E-26	54.95917
50	HLA.DPA1	-1.42543	8.291302	-11.8883	6.96E-29	1.59E-26	54.80154
51	COLEC12	-1.26449	2.900478	-11.8701	8.24E-29	1.82E-26	54.63479
52	EVI2B	-1.04629	4.084858	-11.7678	2.14E-28	4.51E-26	53.69662
53	ADA2	-1.08732	4.772012	-11.7482	2.56E-28	5.30E-26	53.5169
54	CD93	-1.00377	3.746115	-11.7342	2.92E-28	5.97E-26	53.38951
55	ST3GAL5	-1.34501	5.168563	-11.7078	3.73E-28	7.56E-26	53.14805
56	NCKAP1L	-1.06992	3.471657	-11.6652	5.53E-28	1.07E-25	52.76053
57	TNS1	-1.12173	4.764036	-11.586	1.15E-27	2.12E-25	52.04066

58	FMO2	-1.25617	2.97927	-11.567	1.37E-27	2.42E-25	51.86837
59	ALOX5	-1.18774	4.529621	-11.5294	1.93E-27	3.33E-25	51.52814
60	SERPING1	-1.00033	7.727873	-11.4768	3.13E-27	5.13E-25	51.05269
61	CCR7	-1.0423	2.148343	-11.4245	5.04E-27	8.15E-25	50.58202
62	SPI1	-1.11691	4.985115	-11.4159	5.46E-27	8.75E-25	50.50457
63	ACKR1	-1.49378	3.01343	-11.4133	5.59E-27	8.89E-25	50.48113
64	IRF8	-1.05579	3.291536	-11.411	5.71E-27	9.01E-25	50.46064
65	ITGB2	-1.24473	6.206803	-11.3927	6.74E-27	1.04E-24	50.29622
66	CD22	-1.12462	2.141064	-11.3902	6.90E-27	1.06E-24	50.27361
67	ACE	-1.00342	4.138346	-11.3885	7.00E-27	1.06E-24	50.25871
68	CSF1R	-1.12151	4.397087	-11.2945	1.65E-26	2.38E-24	49.41669
69	INMT	-1.47815	3.156856	-11.2767	1.93E-26	2.70E-24	49.25807
70	HMCN1	-1.09713	2.686863	-11.2617	2.22E-26	3.07E-24	49.12418
71	SSC5D	-1.05429	2.733527	-11.2362	2.79E-26	3.80E-24	48.89692
72	MOXD1	-1.1505	3.811641	-11.2253	3.08E-26	4.17E-24	48.79945
73	DPYSL2	-1.00275	5.05104	-11.2148	3.39E-26	4.55E-24	48.7058
74	TMPRSS2	-1.55581	4.939405	-11.201	3.84E-26	5.09E-24	48.58363
75	MEGF6	-1.15593	3.351486	-11.1773	4.76E-26	6.18E-24	48.37256
76	C1orf162	-1.00029	4.442053	-11.164	5.36E-26	6.80E-24	48.25516
77	TRIM22	-1.04032	5.088739	-11.1639	5.36E-26	6.80E-24	48.25396
78	HCLS1	-1.01111	5.095891	-11.1495	6.11E-26	7.55E-24	48.12585
79	CPA3	-1.51197	3.487625	-11.128	7.41E-26	9.01E-24	47.93603
80	CISH	-1.08059	4.554014	-11.1099	8.72E-26	1.04E-23	47.77542
81	HLA.DRA	-1.2943	10.16674	-11.1004	9.50E-26	1.13E-23	47.69151
82	SLC15A3	-1.04694	5.89639	-11.0556	1.42E-25	1.60E-23	47.29542
83	SCN7A	-1.00065	1.564559	-11.0534	1.45E-25	1.63E-23	47.27612
84	AL365361.	-1.08117	1.889931	-10.9965	2.41E-25	2.63E-23	46.77539
85	AQP1	-1.86342	6.681851	-10.9866	2.63E-25	2.86E-23	46.68788
86	SNX30	-1.03312	3.227753	-10.9548	3.49E-25	3.72E-23	46.4095
87	ADH1B	-1.99984	3.385362	-10.9157	4.95E-25	5.14E-23	46.06619
88	RHEX	-1.09672	2.145396	-10.9085	5.28E-25	5.45E-23	46.00328
89	ITGAM	-1.08707	3.022443	-10.8893	6.26E-25	6.31E-23	45.83537
90	TXNIP	-1.06051	7.894655	-10.8828	6.63E-25	6.65E-23	45.77845
91	PLCB2	-1.00097	3.803716	-10.8429	9.44E-25	8.93E-23	45.43037
92	MNDA	-1.08264	3.515298	-10.8427	9.46E-25	8.93E-23	45.42889
93	CCL19	-1.75451	4.832755	-10.7409	2.32E-24	2.06E-22	44.54372
94	MGP	-1.25371	8.099553	-10.7398	2.35E-24	2.06E-22	44.53418
95	MGLL	-1.13547	5.600292	-10.6926	3.55E-24	3.07E-22	44.12635
96	LAIR1	-1.06551	4.432761	-10.6728	4.23E-24	3.63E-22	43.95461
97	C4B	-1.42277	6.346425	-10.6661	4.48E-24	3.83E-22	43.89739
98	CCL14	-1.58529	3.75136	-10.6597	4.74E-24	4.04E-22	43.84165
99	ALOX5AP	-1.22871	5.562996	-10.658	4.81E-24	4.08E-22	43.8272
100	VWF	-1.06475	4.976943	-10.6562	4.89E-24	4.13E-22	43.81145
101	LY86	-1.03018	3.426833	-10.6544	4.97E-24	4.17E-22	43.79657
102	SFTPA2	-3.24124	8.790396	-10.6326	6.01E-24	4.99E-22	43.60879
103	ICAM1	-1.29977	6.994123	-10.5904	8.69E-24	7.08E-22	43.24536
104	DLC1	-1.21452	3.565265	-10.5814	9.40E-24	7.57E-22	43.16807
105	SGPP2	-1.24033	4.425099	-10.5757	9.88E-24	7.92E-22	43.11907
106	HLA.DRB1	-1.44988	8.650994	-10.569	1.05E-23	8.32E-22	43.06147
107	MYH11	-1.42983	3.954354	-10.542	1.33E-23	1.03E-21	42.83036
108	IL7R	-1.15358	3.576347	-10.538	1.37E-23	1.06E-21	42.79615
109	TPSB2	-1.69611	4.664157	-10.5379	1.37E-23	1.06E-21	42.7953
110	TNXB	-1.24805	2.906702	-10.5328	1.44E-23	1.10E-21	42.75141
111	SIGLEC1	-1.02094	2.504853	-10.5306	1.46E-23	1.11E-21	42.7325
112	OLR1	-1.29328	3.580497	-10.5224	1.57E-23	1.19E-21	42.6623
113	HLA.DQB2	-1.84751	4.688924	-10.5215	1.58E-23	1.19E-21	42.65508
114	C1orf116	-1.58709	5.237127	-10.51	1.75E-23	1.31E-21	42.55676
115	C4A	-1.3698	6.320902	-10.4785	2.30E-23	1.67E-21	42.28791
116	CYP27A1	-1.08001	4.833635	-10.4471	3.02E-23	2.12E-21	42.01997

117	LGALS9	-1.03552	6.751306	-10.4441	3.10E-23	2.16E-21	41.99409
118	TLR2	-1.06368	3.445448	-10.4383	3.26E-23	2.25E-21	41.94466
119	VIPR1	-1.08407	2.724182	-10.4293	3.52E-23	2.42E-21	41.86815
120	DCN	-1.18818	7.688772	-10.3909	4.91E-23	3.25E-21	41.54232
121	OSCAR	-1.00085	2.882764	-10.3596	6.43E-23	4.22E-21	41.27687
122	COL14A1	-1.24775	3.285186	-10.3547	6.71E-23	4.37E-21	41.23541
123	GPC4	-1.15763	4.527761	-10.3484	7.08E-23	4.57E-21	41.18197
124	GGTLC1	-2.00185	3.113784	-10.3239	8.74E-23	5.58E-21	40.97492
125	SLC22A31	-1.95596	5.554889	-10.3066	1.01E-22	6.39E-21	40.82869
126	HLA.DQA1	-1.65818	6.875028	-10.3042	1.03E-22	6.50E-21	40.80854
127	SFTPA1	-3.17975	8.339292	-10.2846	1.22E-22	7.54E-21	40.64306
128	SPON1	-1.11049	3.279265	-10.2812	1.26E-22	7.73E-21	40.61451
129	SLCO2A1	-1.17878	3.912497	-10.2588	1.53E-22	9.27E-21	40.42525
130	SPOCK2	-1.07173	3.842701	-10.2265	2.01E-22	1.19E-20	40.15397
131	ABCA3	-1.60369	4.835362	-10.2262	2.02E-22	1.19E-20	40.1516
132	CAVIN2	-1.17587	3.51303	-10.2103	2.31E-22	1.35E-20	40.0178
133	MRC1	-1.36659	3.711277	-10.1536	3.75E-22	2.16E-20	39.54283
134	PTPRC	-1.07803	4.158517	-10.1099	5.43E-22	3.07E-20	39.17861
135	SELL	-1.07373	3.18861	-10.0848	6.72E-22	3.71E-20	38.96894
136	ITIH5	-1.07905	2.118975	-10.0178	1.18E-21	6.28E-20	38.41319
137	LTB	-1.18658	4.390148	-9.97838	1.65E-21	8.56E-20	38.08668
138	HLA.DMB	-1.09728	6.588805	-9.97324	1.72E-21	8.92E-20	38.04417
139	MFSD4A	-1.52663	3.509611	-9.96728	1.81E-21	9.30E-20	37.99492
140	MS4A7	-1.00179	3.899866	-9.96658	1.82E-21	9.34E-20	37.98918
141	FOLR2	-1.1623	4.271689	-9.92482	2.58E-21	1.29E-19	37.64479
142	ATP13A4	-1.61255	3.255137	-9.92151	2.66E-21	1.32E-19	37.6175
143	FAM20A	-1.08214	5.005483	-9.89783	3.24E-21	1.59E-19	37.42272
144	COL8A1	-1.17395	4.661476	-9.88688	3.55E-21	1.73E-19	37.33278
145	KCNK5	-1.18149	3.942215	-9.87949	3.77E-21	1.82E-19	37.27207
146	MARCO	-1.64709	4.618881	-9.86557	4.24E-21	2.04E-19	37.15785
147	TIMP3	-1.12688	6.43284	-9.85045	4.81E-21	2.30E-19	37.03391
148	CD1C	-1.10912	2.070897	-9.84173	5.17E-21	2.46E-19	36.96251
149	DMBT1	-2.21448	3.751612	-9.84109	5.20E-21	2.47E-19	36.95727
150	NKX2.1	-1.57017	5.821701	-9.83346	5.54E-21	2.62E-19	36.89474
151	NFIX	-1.09881	4.164527	-9.8218	6.10E-21	2.87E-19	36.79937
152	SCTR	-1.72949	3.016704	-9.78608	8.21E-21	3.79E-19	36.50763
153	CD52	-1.086	6.794894	-9.75269	1.08E-20	4.89E-19	36.23555
154	ITGBL1	-1.10236	4.214622	-9.71081	1.53E-20	6.80E-19	35.89522
155	CCL22	-1.0621	2.305997	-9.69104	1.80E-20	7.92E-19	35.73492
156	FBLN5	-1.02875	4.357377	-9.6758	2.04E-20	8.92E-19	35.61152
157	FXYD1	-1.14101	2.826121	-9.67176	2.11E-20	9.20E-19	35.57877
158	ISLR	-1.08708	5.018855	-9.60306	3.72E-20	1.57E-18	35.02421
159	MYO1G	-1.20681	3.481139	-9.60193	3.75E-20	1.59E-18	35.01509
160	SLC15A2	-1.05114	2.640739	-9.59946	3.83E-20	1.61E-18	34.99517
161	CEACAM6	-1.9303	8.723896	-9.59368	4.01E-20	1.68E-18	34.94863
162	PIGR	-2.4194	5.98623	-9.58701	4.24E-20	1.77E-18	34.89504
163	CYP2B7P	-1.9531	5.04792	-9.58411	4.34E-20	1.80E-18	34.87169
164	LMO3	-1.60162	4.861895	-9.5708	4.84E-20	2.00E-18	34.76469
165	NDNF	-1.493	4.135809	-9.56686	5.00E-20	2.06E-18	34.733
166	CHIT1	-1.91464	4.490941	-9.52762	6.89E-20	2.78E-18	34.41829
167	C8orf34.AS	-1.18014	1.808444	-9.51887	7.39E-20	2.96E-18	34.34825
168	CDH11	-1.1439	4.693523	-9.50213	8.47E-20	3.36E-18	34.21431
169	SFTA3	-1.79789	6.667144	-9.5021	8.48E-20	3.36E-18	34.21407
170	C16orf89	-2.21841	6.396278	-9.48003	1.01E-19	3.98E-18	34.0378
171	CDC42EP1	-1.03361	5.813262	-9.45585	1.23E-19	4.78E-18	33.84493
172	F13A1	-1.24653	3.504185	-9.43219	1.50E-19	5.74E-18	33.65658
173	DPP4	-1.56559	4.574101	-9.42727	1.56E-19	5.93E-18	33.61746
174	CD1E	-1.05692	1.743991	-9.42538	1.58E-19	6.01E-18	33.60243
175	PTPN13	-1.19779	2.949912	-9.41582	1.71E-19	6.46E-18	33.52646

176	MALL	-1.25252	6.65944	-9.40883	1.81E-19	6.82E-18	33.47099
177	CTSH	-1.24117	8.952259	-9.40081	1.93E-19	7.25E-18	33.40727
178	CXCL12	-1.02407	4.032725	-9.35722	2.74E-19	1.01E-17	33.06199
179	CTSE	-2.27464	5.506812	-9.32253	3.62E-19	1.32E-17	32.78794
180	TREM2	-1.06136	4.574254	-9.32134	3.66E-19	1.33E-17	32.77853
181	CYBB	-1.12031	4.482851	-9.30357	4.22E-19	1.52E-17	32.63848
182	SCNN1B	-1.36667	4.426831	-9.30237	4.26E-19	1.53E-17	32.629
183	RAB7B	-1.09765	3.104985	-9.29066	4.68E-19	1.67E-17	32.53676
184	EFEMP1	-1.08823	5.455919	-9.28952	4.72E-19	1.68E-17	32.5278
185	CFAP221	-1.20163	2.704797	-9.28	5.09E-19	1.80E-17	32.4529
186	FAM189A2	-1.02859	2.530838	-9.2593	6.01E-19	2.10E-17	32.29028
187	SOD3	-1.00025	4.636622	-9.24248	6.88E-19	2.37E-17	32.15829
188	CSF3R	-1.26424	4.270791	-9.1937	1.01E-18	3.36E-17	31.77649
189	HLF	-1.13911	2.124761	-9.11212	1.94E-18	6.02E-17	31.14124
190	MMP2	-1.05891	6.771078	-9.08784	2.35E-18	7.15E-17	30.95295
191	SFTPB	-2.46904	10.59871	-9.05915	2.94E-18	8.83E-17	30.73089
192	COL6A3	-1.04222	6.198938	-9.05522	3.04E-18	9.06E-17	30.70051
193	RASGRF1	-1.30686	2.256959	-9.01578	4.14E-18	1.21E-16	30.3962
194	RNASE1	-1.27488	9.23243	-9.00101	4.65E-18	1.35E-16	30.28245
195	LRRK2	-1.3688	3.023734	-8.96835	6.00E-18	1.72E-16	30.03151
196	NPC2	-1.00455	9.357375	-8.95801	6.51E-18	1.86E-16	29.95219
197	ALOX15B	-1.52689	3.815848	-8.9273	8.27E-18	2.33E-16	29.71692
198	SFTPD	-2.05198	6.161598	-8.90396	9.92E-18	2.76E-16	29.53862
199	DES	-1.17289	2.336145	-8.90341	9.96E-18	2.77E-16	29.53437
200	RHOBTB2	-1.05276	4.74675	-8.89726	1.05E-17	2.90E-16	29.48745
201	XKRX	-1.14836	2.287133	-8.89682	1.05E-17	2.90E-16	29.48411
202	FCGBP	-1.30892	2.545659	-8.82605	1.82E-17	4.83E-16	28.94566
203	ACSL5	-1.08089	5.694252	-8.8208	1.89E-17	5.01E-16	28.90583
204	IL33	-1.01055	3.122688	-8.81812	1.93E-17	5.10E-16	28.88551
205	EGR1	-1.13648	5.811774	-8.80829	2.08E-17	5.49E-16	28.81105
206	FHL1	-1.079	3.715733	-8.79961	2.23E-17	5.84E-16	28.74526
207	SFTA2	-1.69933	8.28793	-8.79509	2.31E-17	6.03E-16	28.71108
208	AGER	-1.7382	4.409595	-8.79359	2.33E-17	6.09E-16	28.69974
209	NRGN	-1.30861	4.173371	-8.77416	2.71E-17	7.02E-16	28.55287
210	C4BPA	-1.92198	5.602401	-8.75412	3.16E-17	8.13E-16	28.40163
211	SCGB1A1	-3.07151	5.644401	-8.74192	3.47E-17	8.90E-16	28.30964
212	B3GNT8	-1.09127	3.497209	-8.7197	4.12E-17	1.05E-15	28.14238
213	AMY1C	-1.86638	4.13237	-8.71964	4.12E-17	1.05E-15	28.14193
214	CHRDL1	-1.14032	2.344765	-8.71759	4.19E-17	1.06E-15	28.12651
215	SUSD2	-1.71444	4.635487	-8.69741	4.88E-17	1.22E-15	27.97497
216	CLEC3B	-1.09342	3.862284	-8.6599	6.51E-17	1.58E-15	27.69386
217	SHE	-1.07649	2.60349	-8.64611	7.23E-17	1.74E-15	27.59075
218	PRODH	-1.6782	4.843949	-8.63601	7.81E-17	1.87E-15	27.51537
219	CAPN8	-1.50718	5.226647	-8.6242	8.54E-17	2.03E-15	27.4272
220	ALPL	-1.56897	4.708794	-8.46475	2.85E-16	6.26E-15	26.24609
221	B3GNT7	-1.21675	4.398691	-8.46182	2.91E-16	6.36E-15	26.22458
222	ATP11A	-1.01567	5.35649	-8.45047	3.17E-16	6.86E-15	26.14115
223	SORCS2	-1.01743	2.124744	-8.4442	3.32E-16	7.16E-15	26.09508
224	MS4A1	-1.24124	2.307679	-8.43593	3.54E-16	7.55E-15	26.03441
225	SFTPC	-3.31529	7.46349	-8.42554	3.82E-16	8.12E-15	25.95819
226	TMEM130	-1.13673	2.130585	-8.38469	5.19E-16	1.07E-14	25.65928
227	PARM1	-1.11825	4.957132	-8.35256	6.59E-16	1.34E-14	25.425
228	CYP4B1	-1.80233	4.35172	-8.33977	7.24E-16	1.46E-14	25.33187
229	RRAD	-1.24885	3.763147	-8.30513	9.37E-16	1.85E-14	25.08028
230	TROAP	1.05064	3.483081	8.298861	9.81E-16	1.93E-14	25.03487
231	C1QC	-1.00221	7.369124	-8.25219	1.38E-15	2.65E-14	24.69739
232	CXCL17	-1.51376	7.729339	-8.249	1.42E-15	2.71E-14	24.67434
233	SCGB3A1	-2.57205	6.693819	-8.22713	1.67E-15	3.14E-14	24.5167
234	MUC21	-1.87282	3.609285	-8.18365	2.29E-15	4.24E-14	24.20429

235	FAXDC2	-1.18168	3.804383	-8.15083	2.91E-15	5.28E-14	23.96935
236	FMO5	-1.19975	4.150938	-8.12923	3.41E-15	6.12E-14	23.81508
237	C1QB	-1.02194	7.757725	-8.12405	3.54E-15	6.34E-14	23.77816
238	LTF	-1.74195	4.427352	-8.12251	3.58E-15	6.40E-14	23.76714
239	SELENBP1	-1.1647	6.523852	-8.10365	4.11E-15	7.30E-14	23.63278
240	CCL18	-1.31226	6.472997	-8.09768	4.29E-15	7.61E-14	23.59037
241	HLA.DRB5	-1.58973	6.617762	-8.09241	4.46E-15	7.88E-14	23.55284
242	AQP3	-1.4505	7.510635	-8.06693	5.36E-15	9.35E-14	23.37193
243	CLIC5	-1.0737	2.514942	-8.05531	5.83E-15	1.01E-13	23.28959
244	POPDC3	1.020576	1.021148	8.046516	6.22E-15	1.07E-13	23.2273
245	TMEM100	-1.01843	2.04689	-8.0326	6.87E-15	1.18E-13	23.12884
246	FDCSP	-1.36202	2.546689	-8.02457	7.28E-15	1.24E-13	23.07214
247	CHI3L2	-1.14109	3.380498	-7.97965	1.01E-14	1.69E-13	22.75549
248	VSIG4	-1.01536	4.675607	-7.97766	1.02E-14	1.71E-13	22.74151
249	SLC26A9	-1.38534	2.838763	-7.97007	1.08E-14	1.80E-13	22.68818
250	S100B	-1.14354	3.313008	-7.95945	1.16E-14	1.93E-13	22.6136
251	ZNF750	-1.02728	2.244023	-7.94106	1.33E-14	2.18E-13	22.48464
252	FCER1A	-1.11715	2.134785	-7.90735	1.69E-14	2.73E-13	22.24877
253	AUTS2	-1.00841	3.760908	-7.89708	1.82E-14	2.92E-13	22.17712
254	HLA.DQB1	-1.42873	6.957651	-7.89157	1.89E-14	3.03E-13	22.13866
255	HOPX	-1.41095	7.62922	-7.86985	2.21E-14	3.51E-13	21.98738
256	LRRN4	-1.21085	2.260865	-7.82729	2.99E-14	4.65E-13	21.69188
257	CPAMD8	-1.10642	2.887579	-7.79314	3.80E-14	5.81E-13	21.45572
258	AQP4	-1.46776	3.358634	-7.78936	3.90E-14	5.97E-13	21.42961
259	CH25H	-1.01443	2.695533	-7.77514	4.32E-14	6.54E-13	21.33148
260	FOLR1	-1.62198	6.611132	-7.75572	4.95E-14	7.40E-13	21.19778
261	LAMP3	-1.10182	4.562768	-7.72742	6.04E-14	8.91E-13	21.00343
262	CXCL14	-1.85933	4.570079	-7.72568	6.11E-14	9.00E-13	20.99148
263	FOS	-1.05625	7.045881	-7.71817	6.44E-14	9.45E-13	20.93996
264	HHIP	-1.06599	1.679177	-7.71468	6.60E-14	9.63E-13	20.91608
265	CCL17	-1.16447	2.475887	-7.64846	1.05E-13	1.47E-12	20.46398
266	C3	-1.14621	8.750138	-7.63811	1.13E-13	1.58E-12	20.39361
267	CLIC6	-1.34913	4.7213	-7.61546	1.32E-13	1.83E-12	20.23983
268	HPGD	-1.45812	4.820617	-7.56988	1.81E-13	2.45E-12	19.9315
269	SLC44A4	-1.10672	5.563147	-7.54265	2.18E-13	2.90E-12	19.74801
270	IGHA2	-1.41138	7.587263	-7.46769	3.65E-13	4.69E-12	19.24554
271	MUC1	-1.10026	9.562518	-7.45882	3.88E-13	4.97E-12	19.18634
272	CD79A	-1.13948	4.443891	-7.45172	4.07E-13	5.19E-12	19.13905
273	ADGRF1	-1.56245	3.64797	-7.3996	5.80E-13	7.21E-12	18.79266
274	HLA.DQA2	-1.22999	3.946506	-7.38417	6.44E-13	7.94E-12	18.69044
275	SFRP4	-1.13065	4.436124	-7.37601	6.81E-13	8.33E-12	18.63647
276	PPP1R1B	-1.63814	4.433337	-7.36431	7.37E-13	8.97E-12	18.55919
277	SFTA1P	-1.40369	4.124931	-7.35784	7.70E-13	9.29E-12	18.51649
278	ADGRD1	-1.00028	3.198302	-7.34057	8.65E-13	1.03E-11	18.40267
279	CHI3L1	-1.24119	6.001164	-7.2955	1.17E-12	1.37E-11	18.10662
280	HABP2	-1.26677	2.485464	-7.28953	1.22E-12	1.42E-11	18.0675
281	KCNN4	-1.15184	4.740226	-7.27307	1.36E-12	1.56E-11	17.95981
282	COL10A1	-1.20205	4.494994	-7.26621	1.43E-12	1.63E-11	17.91501
283	HSD17B6	-1.10136	3.460969	-7.2247	1.88E-12	2.11E-11	17.64456
284	MMP7	-1.48243	5.573554	-7.22334	1.90E-12	2.13E-11	17.63567
285	CACNA2D:	-1.26867	2.987408	-7.21803	1.97E-12	2.20E-11	17.60116
286	LYZ	-1.17752	8.297545	-7.19687	2.26E-12	2.50E-11	17.4639
287	IGHA1	-1.33997	11.05672	-7.17233	2.66E-12	2.92E-11	17.30511
288	KRT16P1	-1.20795	2.2151	-7.1544	3.00E-12	3.24E-11	17.18935
289	CD207	-1.1776	1.930337	-7.14516	3.19E-12	3.43E-11	17.12975
290	STEAP4	-1.08476	4.557539	-7.09486	4.44E-12	4.66E-11	16.80665
291	TMEM163	-1.02286	3.889689	-7.06542	5.39E-12	5.61E-11	16.61835
292	APOD	-1.37858	4.900194	-7.03546	6.55E-12	6.71E-11	16.42746
293	PEBP4	-1.4588	3.438587	-7.01242	7.62E-12	7.69E-11	16.28108

294	WFDC2	-1.12399	9.31504	-6.96504	1.04E-11	1.02E-10	15.98125
295	KRT16P2	-1.30479	2.603533	-6.95309	1.12E-11	1.09E-10	15.90592
296	VSIG2	-1.31115	3.539861	-6.93998	1.22E-11	1.18E-10	15.82338
297	CD1A	-1.00149	1.771947	-6.93952	1.22E-11	1.19E-10	15.82047
298	CRTAC1	-1.13492	2.927195	-6.93032	1.30E-11	1.25E-10	15.76264
299	SCGB3A2	-2.24493	7.295011	-6.9261	1.33E-11	1.28E-10	15.73614
300	LINC00342	-1.04037	2.757021	-6.89636	1.61E-11	1.54E-10	15.54967
301	HAS3	-1.04682	2.988205	-6.86795	1.94E-11	1.82E-10	15.37222
302	KCNJ15	-1.05478	3.341683	-6.84186	2.29E-11	2.12E-10	15.20973
303	SHISA3	-1.2907	1.824508	-6.70615	5.40E-11	4.72E-10	14.37294
304	CCL13	-1.02151	3.748032	-6.67882	6.42E-11	5.53E-10	14.2061
305	CRYM	-1.25895	3.40609	-6.63649	8.36E-11	7.06E-10	13.94882
306	SCEL	-1.01902	3.846164	-6.63632	8.37E-11	7.06E-10	13.94779
307	HAGLR	-1.01175	3.484488	-6.62159	9.17E-11	7.70E-10	13.85857
308	IGHV3.72	-1.12859	3.173034	-6.61257	9.70E-11	8.11E-10	13.80406
309	HLA.DRB6	-1.01906	4.60553	-6.60076	1.04E-10	8.69E-10	13.73273
310	TNC	-1.17013	5.185894	-6.58533	1.15E-10	9.49E-10	13.63967
311	COMP	-1.18115	3.527647	-6.52684	1.65E-10	1.33E-09	13.28878
312	PCP4L1	-1.25025	3.179602	-6.49551	2.00E-10	1.59E-09	13.10188
313	ICAM4	-1.00813	2.682537	-6.48714	2.10E-10	1.67E-09	13.05204
314	FOSB	-1.27797	3.486596	-6.48651	2.11E-10	1.67E-09	13.04832
315	SERPINA1	-1.17609	8.627531	-6.44591	2.70E-10	2.11E-09	12.80753
316	AQP5	-1.7011	3.725769	-6.44048	2.80E-10	2.17E-09	12.77542
317	COL1A1	-1.00654	8.451696	-6.38665	3.87E-10	2.94E-09	12.45838
318	PLA2G2A	-1.03588	2.07445	-6.31573	5.94E-10	4.34E-09	12.04411
319	JCHAIN	-1.06705	8.139991	-6.23661	9.51E-10	6.72E-09	11.58665
320	MMP28	-1.14191	3.475328	-6.20411	1.15E-09	8.09E-09	11.40013
321	HBA2	-1.14105	5.156282	-6.19616	1.21E-09	8.45E-09	11.35462
322	ADIRF	-1.01924	5.225686	-6.19052	1.25E-09	8.71E-09	11.32243
323	PLAT	-1.04559	4.91362	-6.16133	1.48E-09	1.02E-08	11.15593
324	VSTM2L	-1.11837	4.646878	-6.13464	1.73E-09	1.18E-08	11.00431
325	IL37	-1.25696	1.797374	-6.13185	1.76E-09	1.19E-08	10.98847
326	SLC1A7	-1.25346	2.080881	-6.05123	2.82E-09	1.84E-08	10.5341
327	IGHV3.49	-1.26019	4.531345	-6.03892	3.03E-09	1.97E-08	10.46514
328	SLC6A14	-1.0328	3.804642	-5.93521	5.48E-09	3.43E-08	9.889317
329	VSIG1	-1.24431	2.308214	-5.91468	6.16E-09	3.81E-08	9.77633
330	PLA2G1B	-1.05664	2.304625	-5.91169	6.27E-09	3.87E-08	9.759931
331	AGR3	-1.31601	6.00521	-5.90155	6.64E-09	4.07E-08	9.704242
332	HHLA2	-1.27095	2.003399	-5.86648	8.09E-09	4.88E-08	9.512452
333	IGLV2.18	-1.09433	3.122186	-5.85902	8.44E-09	5.07E-08	9.471768
334	IGLV2.8	-1.16854	5.995763	-5.85366	8.70E-09	5.21E-08	9.442589
335	GLB1L3	-1.0852	1.780859	-5.82025	1.05E-08	6.20E-08	9.261081
336	IGHM	-1.21969	8.387557	-5.78791	1.26E-08	7.33E-08	9.086315
337	IGHV3.74	-1.08629	4.761395	-5.75027	1.55E-08	8.90E-08	8.883966
338	IGHD	-1.42873	4.559447	-5.73677	1.67E-08	9.53E-08	8.811665
339	CHIA	-1.08249	2.235352	-5.72335	1.80E-08	1.02E-07	8.739911
340	IGHV3.30	-1.18641	7.389169	-5.68681	2.20E-08	1.23E-07	8.545364
341	CRABP2	-1.28932	6.675527	-5.68307	2.25E-08	1.25E-07	8.525547
342	CST6	-1.02081	3.675819	-5.68202	2.26E-08	1.26E-07	8.519959
343	CLDN18	-1.27549	2.614264	-5.66411	2.49E-08	1.37E-07	8.425063
344	HBA1	-1.00302	4.216313	-5.61033	3.34E-08	1.80E-07	8.14176
345	IGLV7.46	-1.06596	4.334076	-5.57607	4.03E-08	2.13E-07	7.962466
346	IGHV1.2	-1.2141	5.568701	-5.49999	6.06E-08	3.09E-07	7.56787
347	UCHL1	1.248076	4.865695	5.496939	6.16E-08	3.14E-07	7.552162
348	BPIFB1	-1.50248	4.487896	-5.42598	8.99E-08	4.45E-07	7.188635
349	MS4A15	-1.12922	2.280981	-5.41934	9.31E-08	4.60E-07	7.154815
350	IGHG2	-1.07463	9.339911	-5.38273	1.13E-07	5.49E-07	6.969104
351	IGHV5.51	-1.12333	6.49013	-5.31669	1.59E-07	7.53E-07	6.636939
352	IGLV2.11	-1.0578	6.738018	-5.28359	1.89E-07	8.78E-07	6.471842

353	IGHV4.31	-1.09765	4.215622	-5.22649	2.54E-07	1.15E-06	6.189206
354	IGHV3.11	-1.05686	5.649227	-5.19896	2.92E-07	1.31E-06	6.053896
355	HP	-1.38018	4.699214	-5.17287	3.34E-07	1.48E-06	5.926256
356	IGKV2D.29	-1.09357	4.352216	-5.16847	3.41E-07	1.51E-06	5.904779
357	IGLV1.40	-1.00035	7.606215	-5.12863	4.18E-07	1.82E-06	5.711156
358	IGLV1.44	-1.01522	6.904428	-5.08972	5.08E-07	2.17E-06	5.523345
359	IGLV1.51	-1.02804	7.117748	-5.05984	5.90E-07	2.49E-06	5.380025
360	IGHV3.15	-1.00242	5.920674	-4.9521	1.01E-06	4.06E-06	4.869443
361	IGKV1.17	-1.01654	6.293956	-4.94039	1.06E-06	4.28E-06	4.814562
362	IGKV3.20	-1.00561	9.253149	-4.90176	1.28E-06	5.08E-06	4.63435
363	IGHV3.33	-1.09305	6.529594	-4.85151	1.64E-06	6.35E-06	4.401814
364	IGKV1.16	-1.04288	5.897629	-4.8323	1.80E-06	6.91E-06	4.313453
365	CLDN2	-1.05883	2.236636	-4.79135	2.19E-06	8.26E-06	4.126245
366	CST1	-1.14153	3.961672	-4.66157	4.03E-06	1.44E-05	3.54257
367	SNORD3A	1.00332	6.229234	4.655233	4.15E-06	1.48E-05	3.514452
368	AKR1B10	1.287088	2.534204	4.441586	1.10E-05	3.63E-05	2.586831
369	AKR1C2	1.583792	5.03032	4.440033	1.11E-05	3.65E-05	2.580237
370	CEACAM5	-1.27181	6.23074	-4.23395	2.73E-05	8.36E-05	1.723961
371	SPINK1	-1.37212	6.018074	-4.05248	5.87E-05	0.000168	1.001288
372	PGC	-1.42247	5.170011	-3.80187	0.000161	0.000422	0.05217
373	MSLN	-1.08532	6.106722	-3.48357	0.000538	0.001269	-1.07073

Table S4: Kaplan-Meier analyses of 20 relugators in TCGA cohort

	characteris	HR(95%CI)	pvalue
1	APOL1	1.241(1.09	0.000997
2	ATIC	1.464(1.15	0.001964
3	BAK1	1.484(1.15	0.00187
4	BID	1.215(1.03	0.018374
5	BIRC5	1.196(1.07	0.000913
6	CCR2	0.733(0.61	0.00031
7	CDKN1A	1.19(1.004	0.044383
8	DAPK2	0.76(0.651	0.000505
9	DLC1	0.883(0.79	0.02606
10	DRAM1	0.842(0.74	0.009059
11	EIF4G1	1.505(1.19	0.000656
12	FADD	1.699(1.30	9.75E-05
13	GAPDH	1.594(1.33	3.23E-07
14	GNAI3	1.687(1.21	0.001703
15	ITGA6	1.268(1.13	2.70E-05
16	ITGB1	1.59(1.303	4.95E-06
17	ITGB4	1.177(1.06	0.001386
18	PRKCD	0.762(0.62	0.006472
19	RAC1	1.283(1.06	0.009957
20	SPHK1	1.284(1.12	0.000206
